# Comparison of Robot-Assisted and Open Radical Cystectomy in Recovery of Patient-Reported and Performance-Related Measures of Independence: A Secondary Analysis of a Randomized Clinical Trial

**DOI:** 10.1001/jamanetworkopen.2021.48329

**Published:** 2022-02-16

**Authors:** Vivek Venkatramani, Isildinha M. Reis, Mark L. Gonzalgo, Erik P. Castle, Michael E. Woods, Robert S. Svatek, Alon Z. Weizer, Badrinath R. Konety, Mathew Tollefson, Tracey L. Krupski, Norm D. Smith, Ahmad Shabsigh, Daniel A. Barocas, Marcus L. Quek, Atreya Dash, Dipen J. Parekh

**Affiliations:** 1 Department of Urology, University of Miami Miller School of Medicine, Miami, Florida; 2 Sylvester Comprehensive Cancer Center, University of Miami, Miami, Florida; 3 Division of Biostatistics, Department of Public Health Sciences, Miller School of Medicine, University of Miami, Miami, Florida; 4 Department of Urology, Tulane University School of Medicine, New Orleans, Louisiana; 5 Department of Urology, University of North Carolina at Chapel Hill; 6 Department of Urology, Division of Urologic Oncology, University of Texas Health Science Center at San Antonio; 7 Department of Urology, University of Michigan, Ann Arbor; 8 Department of Urology, University of Minnesota, Minneapolis; 9 Department of Urology, Mayo Clinic, Rochester, Minnesota; 10 Department of Urology, University of Virginia Health Science Center, Charlottesville; 11 Department of Urology, North Shore University Health System, Evanston, Illinois; 12 Department of Urology, Ohio State University, Columbus; 13 Department of Urology, Vanderbilt University Medical Center, Nashville, Tennessee; 14 Department of Urology, Loyola University Medical Center, Maywood, Illinois; 15 Department of Urology, University of Washington, Seattle

## Abstract

**Importance:**

No data exist on time to recovery of patient-reported and performance-related measures of functional independence after radical cystectomy (open or robotic).

**Objective:**

To determine recovery of functional independence after radical cystectomy and whether robot-assisted radical cystectomy (RARC) is associated with any advantage over open procedures.

**Design, Setting, and Participants:**

Data for this secondary analysis from the RAZOR (Randomized Open vs Robotic Cystectomy) trial were used. RAZOR was a phase 3 multicenter noninferiority trial across 15 academic medical centers in the US from July 1, 2011, to November 18, 2014, with a median follow-up of 2 years. Participants included the per-protocol population (n = 302). Data were analyzed from February 1, 2017, to May 1, 2021.

**Interventions:**

Robot-assisted radical cystectomy or open radical cystectomy (ORC).

**Main Outcomes and Measures:**

Patient-reported (activities of daily living [ADL] and independent ADL [iADL]) and performance-related (hand grip strength [HGS] and Timed Up & Go walking test [TUGWT]) measures of independence were assessed. Patterns of postoperative recovery for the entire cohort and comparisons between RARC and ORC were performed. Exploratory analyses to assess measures of independence across diversion type and to determine whether baseline impairments were associated with 90-day complications or 1-year mortality were performed.

**Findings:**

Of the 302 patients included in the analysis (254 men [84.1%]; mean [SD] age at consent, 68.0 [9.7] years), 150 underwent RARC and 152 underwent ORC. Baseline characteristics were similar in both groups. For the entire cohort, ADL, iADL, and TUGWT recovered to baseline by 3 postoperative months, whereas HGS recovered by 6 months. There was no difference between RARC and ORC for ADL, iADL, TUGWT, or HGS scores at any time. Activities of daily living recovered 1 month after RARC (mean estimated score, 7.7 [95% CI, 7.3-8.0]) vs 3 months after ORC (mean estimated score, 7.5 [95% CI, 7.2-7.8]). Hand grip strength recovered by 3 months after RARC (mean estimated HGS, 29.0 [95% CI, 26.3-31.7] kg) vs 6 months after ORC (mean estimated HGS, 31.2 [95% CI, 28.8-34.2] kg). In the RARC group, 32 of 90 patients (35.6%) showed a recovery in HGS at 3 months vs 32 of 88 (36.4%) in the ORC group (*P* = .91), indicating a rejection of the primary study hypothesis for HGS. Independent ADL and TUGWT recovered in 3 months for both approaches. Hand grip strength showed earlier recovery in patients undergoing continent urinary diversion (mean HGS at 3 months, 31.3 [95% CI, 27.7-34.8] vs 33.9 [95% CI, 30.5-37.3] at baseline; *P* = .09) than noncontinent urinary diversion (mean HGS at 6 months, 27.4 [95% CI, 24.9-30.0] vs 29.5 [95% CI, 27.2-31.9] kg at baseline; *P* = .02), with no differences in other parameters. Baseline impairments in any parameter were not associated with 90-day complications or 1-year mortality.

**Conclusions and Relevance:**

The results of this secondary analysis suggest that patients require 3 to 6 months to recover baseline levels after radical cystectomy irrespective of surgical approach. These data will be invaluable in patient counseling and preparation. Hand grip strength and ADL tended to recover to baseline earlier after RARC; however, there was no difference in the percentage of patients recovering when compared with ORC. Further study is needed to assess the clinical significance of these findings.

**Trial Registration:**

ClinicalTrials.gov Identifier: NCT01157676

## Key Points

**Question:**

Is robot-assisted radical cystectomy (RARC) associated with faster recovery of patient-reported and performance-related measures of independence when compared with open radical cystectomy (ORC)?

**Findings:**

This secondary analysis of the RAZOR (Randomized Open vs Robotic Cystectomy) trial found that patients require 3 to 6 months to recover baseline levels of independence irrespective of surgical approach. Hand grip strength and activities of daily living recovered earlier after RARC; however, the percentage of patients recovering at each time point was similar in both groups.

**Meaning:**

These findings suggest that recovery after radical cystectomy takes 3 to 6 months and recovery in some domains may be quicker after RARC; however, further study is needed to assess the clinical significance of these findings.

## Teaser

This secondary analysis of a randomized clinical trial evaluated patient-reported and performance-related measures of independence in patients undergoing robot-assisted vs open radical cystectomy.

## Introduction

Robotic surgery is the foremost minimally invasive surgical alternative in urologic oncology. Initial studies of robot-assisted radical cystectomy (RARC) focused on proving oncologic safety, and in prior publications, including the RAZOR (Randomized Open vs Robotic Cystectomy) trial,^1,2^ this has been well established. Radical cystectomy remains the standard treatment of muscle-invasive bladder carcinoma; however, complete recovery may take months. Despite decades of experience, there remains a paucity of data regarding quality of life (QOL) and patient recovery outcomes. Analysis from the RAZOR trial revealed no significant difference between RARC and open radical cystectomy (ORC) in QOL outcomes, with QOL taking 3 to 6 months to recover after surgery.^3^

Comparative studies between RARC and ORC focus on short-term outcomes such as complications and readmission rates. Robotic-assisted radical cystectomy was developed to quicken patient recovery and reduce procedural morbidity, and a large proportion of patients undergoing radical cystectomy are elderly. To our knowledge, no data exist on the time to recovery of patient-reported and performance-related measures of functional independence after RARC, and it is essential to study whether RARC is associated with any advantage in this regard. We evaluated these measures of independence in patients from the RAZOR trial, which compared RARC and ORC.^4^ Patient-reported outcomes included activities of daily living (ADL) and instrumental ADL (iADL). Performance-related measures included hand grip strength (HGS) and the Timed Up & Go walking test (TUGWT).

## Methods

RAZOR was a phase 3, open-label noninferiority randomized clinical trial across 15 academic medical centers in the US from July 1, 2011, to November 18, 2014. The complete trial protocol is found in Supplement 1. Institutional review board approval was obtained at each site. Written informed consent was obtained from all participants. The Consolidated Standards of Reporting Trials (CONSORT) guidelines for randomized clinical trials were followed for trial publication (eFigure 4 in Supplement 2).^1^ The present analysis was a secondary analysis of the per-protocol population of the RAZOR trial.^4^

Patient-reported measures included ADL and iADL. Performance-related measures included HGS and the TUGWT. These were administered by a clinical research nurse to study participants at baseline, discharge from the hospital, and 4 to 6 weeks (1 month), 3 months, and 6 months after surgery. Surgeons were blinded to the results.

Activities of daily living included 7 items: grooming, feeding, toilet use, bathing, dressing, transferring from bed to chair, and ambulating across a room. Instrumental ADL included 8 additional, more complex self-care abilities: using the telephone, accessing transportation away from home, purchasing groceries, preparing meals, housework, laundry, managing medication, and managing finances. Reverse 3-point scales of no help needed (1 point), needing help (2 points), and unable to do (3 points) were used to score the activities. Missing activities or activities irrelevant to the patient and therefore not scored were replaced with a mean of the other scored items. Activities of daily living scores of 7 and 21 indicate full independence and complete dependence, respectively; iADL scores of 8 and 24, full independence and complete dependence, respectively.

In the TUGWT, patients are timed as they stand from a chair, walk 3 m, turn, walk back, and sit again. This test combines coordination, balance, lower extremity strength for arising from a chair, and the ability to turn and has been shown to correlate well with functional capacity. Values are measured in seconds, and higher values represent worse scores.

Hand grip strength was measured in kilograms of pressure using a handheld dynamometer. A maximum of 4 trials was allowed. Values for HGS are given in kilograms, thus, higher values are considered better. Hand grip strength at 3 months after surgery was used as a surrogate for postoperative recovery.

### Statistical Analysis

Data were analyzed from February 1, 2017, to May 1, 2021. Using measurement of HGS at 3 months after surgery as a surrogate for assessing recovery, patients were categorized as not recovered or recovered to (or better than) preoperative HGS. The study hypothesis was that 20% more patients would have recovered their HGS 3 months after surgery in the RARC group compared with the ORC group. For power calculation, we assumed that 39% of patients would recover HGS at 3 months as reported by Lawrence et al.^5^ A total of 288 patients yielded 91% power at a 1-sided significance level of .025 to detect an improvement of at least 20%, using a *z* test for comparison of 2 independents proportions.

For each functional outcome, we performed mixed-modeling repeated-measures analysis using residual maximum likelihood estimation and assuming any missing data are missing at random. The association of surgical group, urinary diversion, and time after surgery with each outcome was evaluated, and models included adjustment for 6 fixed-effect covariates: age (continuous), sex, body mass index (calculated as weight in kilograms divided by height in meters squared [<25.0, 25.0-29.9, and ≥30.0]), Eastern Cooperative Oncology Group Performance Status (0 or ≥1), T stage (Ta, Tis, and T1-T2 or T3-T4), and perioperative chemotherapy (no or yes). Time was considered a fixed effect, and we assumed a heterogeneous autoregressive covariance matrix to account for the correlated data structure. We included random intercepts for sites with participants nested within sites and assumed variance-covariance unstructured, modeling a different variance component for each site. We also included group × diversion, time × group, and time × diversion interactions in these models regardless of their significance to allow estimation of means of summary scores by group and by diversion type over time. For each functional outcome, we report estimated means of summary score with corresponding 95% CIs. Two-sided *P* values for pairwise comparisons were adjusted for multiple comparisons using the Bonferroni method, with statistical significance set at *P* ≤ .05.

We tested for associations between group, urinary diversion, or a binary baseline functional outcome and complications (including any [grades I-V] and major [grades III-V]) within 90 days from surgery, using the χ^2^ test or the Fisher exact test. Complications were graded using the modified Clavien-Dindo classification.^6^ Kaplan-Meier estimates of the postoperative 1-year mortality rates by group, diversion, and the same baseline functional outcomes were compared using the cloglog transform test.^7^ Baseline summary score values of ADL, iADL, and TUGWT were grouped as normal or abnormal using cut points equivalent to those used by Chesnut et al.^8^ Using the reverse 3-point scales for ADL and iADL, a lower score related to better independence, with normal defined by an ADL score of 7 and an iADLscore of 8, corresponding to no help needed in all items. For the TUGWT, we used a cutoff of less than 10 seconds for normal; for HGS we report below and above median. Data analyses were performed using SAS, version 9.3 (SAS Institute, Inc).

## Results

The RARC group included 150 patients and the ORC group included 152 patients (total of 302 patients; 254 men [84.1%] and 48 women [15.9%]; mean [SD] age at consent, 68.0 [9.7] years). Baseline characteristics including age, sex, performance status, body mass index, tumor stage, diversion type, and receipt of perioperative chemotherapy were similar in both groups (Table 1).

**Table 1.** Patient Characteristics by Treatment Group and Type of Urinary Diversion^a^

Characteristic	Treatment group			*P* value	Urinary diversion^b^		*P* value
	All (N = 302)	RARC (n = 150)	ORC (n = 152)		CUD (n = 67)	NCUD (n = 235)	
Treatment group							
RARC	150 (49.7)	150 (100)	0	NA	37 (55.2)	113 (48.1)	.30
ORC	152 (50.3)	0	152 (100)		30 (44.8)	122 (51.9)	
Type of diversion							
CUD	67 (22.2)	37 (24.7)	30 (19.7)	.30	67 (100)	0	NA
NCUD	235 (77.8)	113 (75.3)	122 (80.3)		0	235 (100)	
Age at consent, y							
Mean (SD)	68.X (9.7)	68.6 (10.3)	67.5 (9.0)	.32	58.2 (7.8)	70.8 (8.3)	<.001
Median (range)	69 (37-90)	70 (43-90)	67 (37-85)		58 (37-79)	71 (43-90)	
Sex							
Men	254 (84.1)	126 (84.0)	128 (84.2)	.96	55 (82.1)	199 (84.7)	.61
Women	48 (15.9)	24 (16.0)	24 (15.8)		12 (17.9)	36 (15.3)	
BMI							
<25.0	77 (25.5)	38 (25.3)	39 (25.7)	.90	19 (28.4)	58 (24.7)	.43
25.0-29.9	124 (41.1)	60 (40.0)	64 (42.1)		30 (44.8)	94 (40.0)	
≥30.0	101 (33.4)	52 (34.7)	49 (32.2)		18 (26.9)	83 (35.3)	
ECOG performance status							
0	226 (74.8)	117 (78.0)	109 (71.7)	.21	56 (83.6)	170 (72.3)	.06
≥1	76 (25.2)	33 (22.0)	43 (28.3)		11 (16.4)	65 (27.7)	
T stage							
Ta, Tis, and T1-T2	262 (86.8)	130 (86.7)	132 (86.8)	.96	58 (86.6)	204 (86.8)	.96
T3-T4	40 (13.2)	20 (13.3)	20 (13.2)		9 (13.4)	31 (13.2)	
Perioperative chemotherapy							
Yes	132 (43.7)	61 (40.7)	71 (46.7)	.29	32 (47.8)	100 (42.5)	.49
No	170 (56.3)	89 (59.3)	81 (53.3)		35 (52.2)	135 (57.4)	
Neoadjuvant chemotherapy							
Yes	97 (32.1)	41 (27.3)	56 (36.8)	.08	26 (38.8)	71 (30.2)	.18
No	205 (67.9)	109 (72.7)	96 (63.2)		41 (61.2)	164 (69.8)	
Adjuvant chemotherapy							
Yes	42 (13.9)	25 (16.7)	17 (11.2)	.17	7 (10.4)	35 (14.9)	.35
No	260 (86.1)	125 (83.3)	135 (88.8)		60 (89.6)	200 (85.1)	
Complications within 90 d, grade							
III-V	67 (22.2)	33 (22.0)	34 (22.4)	.94	12 (17.9)	55 (23.4)	.34
0-II	235 (77.8)	117 (78.0)	118 (77.6)		55 (82.1)	180 (76.6)	

Abbreviations: BMI, body mass index (calculated as weight in kilograms divided by height in meters squared); CUD, continent urinary diversion; ECOG, Eastern Cooperative Oncology Group; NA, not applicable; NCUD, non-CUD; ORC, open radical cystectomy; RARC, robot-assisted radical cystectomy.

^a^ Unless otherwise indicated, data are expressed as the number (%) of patients. Percentages have been rounded and may not total 100.

^b^ Includes 67 neobladder and 1 continent cut reservoir for CUD and 235 conduit for NCUD.

In the combined cohort, ADL was significantly worse at 1 month compared with baseline (estimated mean score 7.8 [95% CI, 7.5-8.1] vs 7.3 [95% CI, 7.1-7.5]; *P* = .02) but patients recovered by 3 months. Instrumental ADL was significantly worse at 1 month compared with baseline (estimated mean score, 11.1 [95% CI, 10.4-11.7] vs 9.2 [95% CI, 8.7-9.6]; *P* < .001) but patients recovered by 3 months. Similarly, TUGWT was significantly worse at 1 month compared with baseline in patients (estimated mean score, 14.4 [95% CI, 12.5-16.4] vs 12.7 [95% CI, 10.8-14.7]; *P* < .001) and recovered by 3 months. The HGS score was significantly worse at 3 months compared with baseline (estimated mean score, 28.9 [95% CI, 26.5-31.4] vs 31.7 [95% CI, 29.3-34.2]; *P* < .001) and recovered by 6 months (Table 2 and eFigure 1 in Supplement 2).

**Table 2.** Patient-Reported and Performance-Related Summary Scores of the Entire Cohort^a^

Measure	Baseline	Postoperative month		
		1	3	6
ADL^b^				
No. of patients	271	248	214	206
Estimated score, mean (95% CI)	7.3 (7.1-7.5)	7.8 (7.5-8.1)^c^	7.4 (7.2-7.7)	7.3 (7.1-7.5)
iADL^d^				
No. of patients	268	242	209	203
Estimated score, mean (95% CI)	9.2 (8.7-9.6)	11.1 (10.4-11.7)^c^	9.6 (9.1-10.1)	9.2 (8.7-9.7)
TUGWT, s^e^				
No. of patients	247	198	166	162
Estimated, mean (95% CI)	12.7 (10.8-14.7)	14.4 (12.5-16.4)^c^	12.7 (10.8-14.6)	12.5 (10.6-14.4)
HGS, kg^f^				
No. of patients	259	216	178	172
Estimated, mean (95% CI)	31.7 (29.3-34.2)	28.9 (26.5-31.3)^c^	28.9 (26.5-31.4)^c^	30.5 (27.9-33.1)

Abbreviations: ADL, activities of daily living; HGS, hand grip strength; iADL, instrumental activities of daily living; TUGWT, Timed Up & Go walking test.

^a^ Estimated means and 95% CIs are calculated from mixed models including time, group, diversion, time × group and time × diversion interactions, age, sex, body mass index, Eastern Cooperative Oncology Group performance status, T stage, and perioperative chemotherapy and accounting for site in the random-effect component of the model.

^b^ Scores range from 7 to 21, with higher scores indicating greater disability.

^c^ *P* ≤ .05, mean difference compared with baseline, statistically significant at the 5% significance using the Bonferroni adjustment for multiple comparisons.

^d^ Scores range from 8 to 24, with higher scores indicating greater disability.

^e^ Higher scores indicate worse performance.

^f^ Higher scores indicate better performance.

In the RARC group, 32 of 90 patients (35.5%) showed a recovery in HGS at 3 months vs 32 of 88 (36.4%) in the ORC group (*P* = .91), indicating a rejection of the primary study hypothesis for HGS. There was also no difference between groups with respect to percentage of patients who show recovery in HGS at 1 month (39 of 104 [37.5%] in RARC vs 48 of 112 [42.9%] in ORC; *P* = .42) (eTable in Supplement 2).

There was no significant difference between RARC and ORC for ADL, iADL, TUGWT, or HGS at any time point (Table 3 and eFigure 2 in Supplement 2). Estimated mean ADL score at 1 month was significantly higher than baseline only in ORC (7.9 [95% CI, 7.5-8.2] vs 7.3 [95% CI, 7.1-7.6]; *P* = .001). In RARC, the difference between estimated mean ADL score at 1 month and baseline was not statistically significant (7.7 [95% CI, 7.3-8.0] vs 7.3 [95% CI, 7.1-7.6]; *P* = .07). However, there was no significant difference between groups with respect to the percentage of recovered patients at 1 month after surgery (98 of 123 [79.7%] in RARC vs 95 of 125 [76.0%] in ORC; *P* = .49) (eTable in Supplement 2).

**Table 3.** Comparison of Patient-Reported and Performance-Related Summary Scores by Treatment Group^a^

Measure by treatment group	Baseline	Postoperative month		
		1	3	6
ADL^b^				
RARC				
No. of patients	133	123	109	103
Estimated score, mean (95% CI)	7.3 (7.1-7.6)	7.7 (7.3-8.0)	7.4 (7.1-7.7)	7.3 (7.1-7.5)
ORC				
No. of patients	138	125	105	103
Estimated score, mean (95% CI)	7.3 (7.1-7.6)	7.9 (7.5-8.2)^c^	7.5 (7.2-7.8)	7.2 (7.0-7.5)
iADL^d^				
RARC				
No. of patients	132	120	105	101
Estimated score, mean (95% CI)	9.0 (8.5-9.5)	10.8 (10.0-11.6)^g^	9.4 (8.8-10.0)	9.2 (8.7-9.8)
ORC				
No. of patients	136	122	104	102
Estimated score, mean (95% CI)	9.4 (8.9-9.9)	11.3 (10.5-12.1)^c^	9.8 (9.1-10.4)	9.2 (8.6-9.7)
TUGWT, s^e^				
RARC				
No. of patients	122	96	83	81
Estimated, mean (95% CI)	12.3 (10.3-14.3)	14.0 (11.9-16.0)^g^	12.1 (10.1-14.0)	12.0 (9.9-14.0)
ORC				
No. of patients	125	102	83	81
Estimated, mean (95% CI)	13.2 (11.1-15.2)	14.9 (12.8-17.0)^c^	13.4 (11.4-15.4)	13.0 (10.9-15.1)
HGS, kg^f^				
RARC				
No. of patients	127	104	90	86
Estimated, mean (95% CI)	31.1 (28.4-33.8)	28.7 (26.0-31.3)^g^	29.0 (26.3-31.7)	29.8 (26.9-32.7)
ORC				
No. of patients	132	112	88	86
Estimated, mean (95% CI)	32.4 (29.7-35.1)	29.2 (26.5-31.9)^c^	28.8 (26.3-31.6)^c^	31.2 (28.2-34.2)

Abbreviations: ADL, activities of daily living; HGS, hand grip strength; iADL, instrumental activities of daily living; ORC, open radical cystectomy; RARC, robot-assisted radical cystectomy; TUGWT, Timed Up & Go walking test.

^a^ The estimated means and 95% CIs are calculated from mixed models including time, group, diversion, time × group and time × diversion interactions, age, sex, body mass index, Eastern Cooperative Oncology Group performance status, T stage, and perioperative chemotherapy and accounting for site in the random-effect component of the model.

^b^ Scores range from 7 to 21, with higher scores indicating greater disability.

^c^ *P* ≤ .05, mean difference compared with baseline, statistically significant at the 5% significance using the Bonferroni adjustment for multiple comparisons.

^d^ Scores range from 8 to 24, with higher scores indicating greater disability.

^e^ Higher scores indicate worse performance.

^f^ Higher scores indicate better performance.

^g^ *P* ≤ .05, mean difference compared with baseline, statistically significant at the 5% significance using the Bonferroni adjustment for multiple comparisons.

The estimated mean iADL score at 1 month was significantly higher than at baseline in the RARC group (10.8 [95% CI, 10.0-11.6] vs 9.0 [95% CI, 8.5-9.5]; *P* < .001) and the ORC group (11.3 [95% CI, 10.5-12.1] vs 9.4 [95% CI, 8.9-9.9]; *P* < .001). Also, there was a significant increase in the mean estimated TUGWT at 1 month vs baseline for the RARC group (14.0 [95% CI, 11.9-16.0] vs 12.3 [95% CI, 10.3-14.3] seconds; *P* = .01) and the ORC group (14.9 [95% CI, 12.8-17.0] vs 13.2 [95% CI, 11.1-15.2] seconds; *P* = .01). These parameters returned to baseline by 3 months.

In the RARC group, there was a statistically significant decrease of estimated mean HGS from baseline (31.1 [95% CI, 28.4-33.8] kg) at 1 month (28.7 [95% CI, 26.0-31.3] kg; *P* = .005), but it returned to baseline at 3 months (29.0 [95% CI, 26.3-31.7] kg; *P* = .25). In the ORC group, there was a statistically significant decrease of estimated mean HGS from baseline (32.4 [95% CI, 29.7-35.1] kg) at 1 month (29.2 [95% CI, 26.5-31.9] kg; *P* = .001) and 3 months (28.8 [95% CI, 26.3-31.6] kg; *P* = .0025), suggesting a slightly delayed return to baseline when compared with RARC; however, as noted above, the proportion of patients who recovered at 3 months was not significantly different (Table 3 and eFigure 2 in Supplement 2).

In the per-protocol population, 67 patients (22.2%) had a continent urinary diversion (CUD) (37 of 150 [24.7%] in the RARC group and 30 of 152 [19.7%] in the ORC group), and 235 (77.8%) had a non-CUD (NCUD) (113 of 150 [75.3%] in the RARC group and 122 of 152 [80.3%] in the ORC group). All parameters showed no difference between CUD and NCUD except HGS at 6 months (estimated mean, 33.6 [95% CI, 29.8-37.3] vs 27.4 [95% CI, 24.9-30.0] kg for NCUD; *P* = .02) (Table 4 and eFigure 3 in Supplement 2). Hand grip strength returned to baseline by 3 months in CUD (mean HGS at 3 months, 31.3 [95% CI, 27.7-34.8] vs 33.9 [95% CI, 30.5-37.3] at baseline; *P* = .34) but had not returned to baseline even at 6 months in NCUD (mean HGS at 6 months as shown above vs 29.5 [95% CI, 27.2-31.9] kg at baseline; *P* = .01). Activities of daily living and TUGWT showed no deterioration from baseline after surgery in patients undergoing CUD. Instrumental ADL returned to baseline at 3months after CUD versus 6 months after NCUD (Table 4 and eFigure 3 in Supplement 2). Analysis of 90-day complications and 1 year mortality (Table 5) did not reveal significantly higher rates for patients with baseline impairments in ADL, iADL, TUGWT, and HGS.

**Table 4.** Comparison of Patient-Reported and Performance-Related Summary Scores by Type of Urinary Diversion^a^

Measure by urinary diversion	Baseline	Postoperative month		
		1	3	6

ADL^b^				
CUD				
No. of patients	63	58	50	48
Estimated score, mean (95% CI)	7.3 (6.9-7.6)	7.5 (7.0-8.0)	7.2 (6.8-7.6)	7.1 (6.8-7.4)
NCUD				
No. of patients	208	190	164	158
Estimated score, mean (95% CI)	7.4 (7.2-7.6)	8.0 (7.7-8.3)^c^	7.7 (7.4-7.9)^c^	7.4 (7.2-7.6)
iADL^d^				
CUD				
No. of patients	62	57	48	47
Estimated score, mean (95% CI)	9.0 (8.4-9.7)	10.8 (9.7-11.9)^c^	9.1 (8.3-9.9)	8.8 (8.1-9.5)
NCUD				
No. of patients	206	185	161	156
Estimated score, mean (95% CI)	9.3 (8.9-9.8)	11.3 (10.7-11.9)^c^	10.1 (9.6-10.6)^c^	9.7 (9.2-10.1)
TUGWT, s^e^				
CUD				
No. of patients	61	49	40	39
Estimated, mean (95% CI)	12.8 (10.4-15.1)	14.5 (12.1-16.9)	12.2 (9.8-14.5)	11.8 (9.3-14.2)
NCUD				
No. of patients	186	149	126	123
Estimated, mean (95% CI)	12.7 (10.9-14.6)	14.4 (12.5-16.3)^c^	13.3 (11.4-15.1)	13.2 (11.3-15.1)
HGS (higher is best), kg^f^				
CUD				
No. of patients	63	53	42	41
Estimated, mean (95% CI)	33.9 (30.5-37.3)	30.5 (27.1-33.9)^c^	31.3 (27.7-34.8)	33.6 (29.8-37.3)^g^
NCUD				
No. of patients	196	163	136	131
Estimated, mean (95% CI)	29.5 (27.2-31.9)	27.3 (25.0-29.7)^c^	26.6 (24.2-29.0)^c^	27.4 (24.9-30.0)^g^

Abbreviations: ADL, activities of daily living; CUD, continent urinary diversion; HGS, hand grip strength; iADL, independent activities of daily living; NCUD, non-CUD; TUGWT, Timed Up & Go walking test.

^a^ Estimated means and 95% CIs are calculated from mixed models including time, group, diversion, time × group and time × diversion interactions, age, sex, body mass index, Eastern Cooperative Oncology Group performance status, T stage, and perioperative chemotherapy and accounting for site in the random-effect component of the model. CUD includes 67 neobladder and 1 continent cut reservoir; NCUD, 235 conduit.

^b^ Scores range from 7 to 21, with higher scores indicating greater disability.

^c^ *P* ≤ .05: Mean difference compared with baseline, statistically significant at the 5% significance using the Bonferroni adjustment for multiple comparisons.

^d^ Scores range from 8 to 24, with higher scores indicating greater disability.

^e^ Higher scores indicate worse performance.

^f^ Higher scores indicate better performance.

^g^ Adjusted *P* = .02 for CUD minus NCUD HGS mean difference of 6.2 kg.

**Table 5.** Rates of Complications Within 90 Days of Radical Cystectomy and 1-Year Cumulative Mortality Rate

Measure or group	No. of patients^a^	Complications within 90 d from surgery				Deaths within 1 y from surgery	
		Any (grades I-V)		Major (grades III-V)			
		No. (%)	*P* value	No. (%)	*P* value	No. of events (cumulative mortality rate, %)	*P* value
Entire cohort	302	206 (68.2)		67 (22.2)		39 (12.9)	NA
Treatment group							
RARC	150	101 (67.3)	.75	33 (22.0)	.94	15 (10.1)	.12
ORC	152	105 (69.1)		34 (22.4)		24 (16.2)	
Type of urinary diversion							
CUD	235	156 (66.4)	.20	55 (23.4)	.34	36 (15.6)	.012
NCUD	67	50 (74.6)		12 (17.9)		3 (4.6)	
Baseline ADL^b^							
Abnormal (>7)	18	11 (61.1)	.60	2 (11.1)	.54	3 (16.7)	.48
Normal (7)	253	170 (67.2)		50 (19.8)		27 (10.8)	
Baseline iADL^c^							
Abnormal (>8)	43	30 (69.8)	.61	13 (30.2)	.06	7 (16.5)	.23
Normal (8)	225	148 (65.8)		38 (16.9)		22 (9.9)	
Baseline TUGWT, s^d^							
Abnormal (≥10)	182	120 (65.9)	.63	38 (20.9)	.68	20 (11.1)	.47
Normal (<10)	65	45 (69.2)		12 (18.5)		5 (7.8)	
Baseline HGS, kg^e^							
≤36 (median)	129	87 (67.4)	.93	28 (21.7)	.62	17 (13.3)	.22
>36	130	87 (66.9)		25 (19.2)		11 (8.5)	

Abbreviations: ADL, activities of daily living; CUD, continent urinary diversion; HGS, hand grip strength; iADL, instrumental activities of daily living; NA, not applicable; NCUD, non-CUD; ORC, open radical cystectomy; RARC, robot-assisted radical cystectomy; TUGWT, Timed Up & Go walking test.

^a^ Sample size differences are due to availability of data.

^b^ Scores range from 7 to 21, with higher scores indicating greater disability.

^c^ Scores range from 8 to 24, with higher scores indicating greater disability.

^d^ Higher scores indicate worse performance.

^e^ Higher scores indicate better performance.

## Discussion

To the best of our knowledge, this report represents the first data on patient-reported and performance-related measures of independence and their recovery patterns after RARC and ORC from a prospective randomized clinical trial. This study demonstrates a worsening of independence measures after radical cystectomy, with recovery taking 3 to 6 months after surgery. There was no significant difference in domain scores between patients undergoing RARC and ORC. Activities of daily living and HGS showed a quicker recovery to baseline in the RARC group (at 1 and 3 months, respectively) vs ORC (3 and 6 months, respectively). Although this difference could indicate a potential benefit for patients undergoing RARC, the percentage of patients recovering in each group was similar at each time point, and the null hypothesis for HGS was rejected. This finding reemphasizes the need for further appropriately designed trials regarding these domains with a view to setting realistic expectations before radical cystectomy.

Lawrence et al^5^ published the seminal report on recovery after abdominal surgery in patients older than 60 years, but this included only 1 patient who underwent radical cystectomy. Most patients underwent colorectal, aortic, and upper abdominal surgery. No minimally invasive cases were included. The mean (SD) age of patients was 69 (6) years, which is similar to that of our study. In their cohort of 372 patients, recovery to baseline took a mean of 6 weeks for TUGWT, 3 months for ADL, and 6 months for iADL, whereas HGS had not returned at 6 months after surgery.^5^ Lawrence et al^5^ concluded that the risk of disability even at 6 months after major surgery remained significant, and potentially modifiable factors, including preoperative physical status, depression, and postoperative complications, were associated with recovery.^5^

A recent prospective study by Osterman et al^9^ assessed recovery patterns of a number of domains after radical cystectomy, with a focus on patients older than 70 years. In both older and younger patients, the investigators observed worsening of TUGWT and iADL at 1 month after radical cystectomy with recovery by 3 months. Older patients were observed to have a greater worsening in physical function and QOL at 1 month.^9^

McMullen et al^10^ also reported that self-care was significantly affected after surgery, and transition to the home imposed a significant challenge. They used focus groups to elicit patient perspectives regarding preoperative decision making and complications of and recovery from radical cystectomy and provided a framework for assessing these domains. Our study offers high-quality prospective data on the recovery of a number of these domains.

Activities of daily living and iADL are widely used to quantify patient independence and are often used in geriatric research.^11^ In the geriatric population, ADL correlates with QOL, readmission rates within 30 days, and mortality.^12,13,14^ In urology, ADL has been shown to be independently associated with complications after percutaneous nephrolithotomy with a greater accuracy than the Charlson Comorbidity Index.^15^ Murray et al^16^ studied ADL in 471 patients in nursing homes who underwent radical cystectomy. All patients showed a worsening of ADL after surgery with the worst performance in bed mobility, transfers, and locomotion in the unit. Extensive physical assistance was needed with transfer, dressing, and toilet use after radical cystectomy. Most patients who had a preoperative assessment for comparison returned to baseline by 3 months after radical cystectomy, which is similar to findings in our study. The previous study included only patients managed in nursing homes after radical cystectomy, and the mean age of patients was 80.7 years. The authors suggested that the increased dependence of elderly patients after radical cystectomy should be discussed with patients and their families.^16^

The TUGWT has been shown to correlate well with functional capacity.^5^ In a study by Chesnut et al^8^ of 65 patients older than 75 years undergoing radical cystectomy, a rapid electronic fitness assessment was used that included ADL, iADL, and TUGWT, among other domains. Patients with baseline impairments in ADL, iADL, or TUGWT were more likely to be admitted to the intensive care unit postoperatively and less likely to be discharged home; however, recovery patterns of these parameters were not reported. Major complications at 30 days were more likely in patients with impairments in TUGWT, whereas 90-day major complications were more likely with impairments in iADL or TUGWT. Ninety-day mortality was also more likely among patients with deficits in ADL, iADL, and TUGWT.^8^ These data underscore the importance of assessment of baseline measures of performance in patients undergoing radical cystectomy. However, the previous study was small, with insufficient statistical power. Our study showed no correlation between baseline impairments in these parameters and complication or mortality rates. Further trials specifically powered to look at these outcomes are needed to improve our understanding and potentially allow preoperative classification and targeted interventions in higher-risk groups.

Baseline HGS has been shown to correlate with nutritional and functional status and to be associated with disability or death in healthy men, whereas failure of recovery of HGS at 1 week may portend postoperative complications.^5,17,18,19^ Isoyama et al^20^ demonstrated that worse HGS is independently associated with mortality among patients undergoing dialysis. To our knowledge, our study includes the first reported data on HGS and its recovery after radical cystectomy. Our patients recovered by 6 months, and although HGS recovered earlier after RARC, the clinical importance of this finding needs further study. We also found no increase in 1-year mortality among patients with lower baseline HGS.

Prehabilitation refers to optimization of nutrition combined with a preoperative exercise regimen in patients before undergoing radical cystectomy. Previous studies have shown the feasibility of this approach and have reported improvements in patient endurance, QOL, and functional recovery.^21,22^ The potential effects of prehabilitation on HGS and its ability to modify the morbidity of RC merit further study.

### Limitations

This study has some limitations. We describe the results of a post hoc analysis of the RAZOR trial, which was not specifically powered to determine significant differences between RARC and ORC (except HGS). Incomplete postoperative data collection was also observed and could have affected the results. However, we believe these data will be valuable in patient counseling and preparation before surgery, but appropriately designed studies will be needed to reach definitive conclusions.

The significantly better HGS at 6 months in patients undergoing CUD vs NCUD and the more rapid recovery of patients undergoing CUD could point to the fact that generally fitter and more independent patients are chosen for CUD. In the RAZOR trial, diversion type was not randomized, and a selection bias was likely among the patients chosen for CUD, accounting for the differences in recovery patterns noted. No data comparing these end points across diversion types exist in the literature to date, and appropriately designed studies comparing different diversions are needed to reach definitive conclusions.

## Conclusions

The findings of this secondary analysis of the RAZOR trial suggest that measures of independence require 3 to 6 months to return to baseline after radical cystectomy irrespective of surgical approach and type of urinary diversion. There was no significant difference in scores between the RARC and ORC groups. Patient HGS and ADL scores returned to baseline values earlier after RARC, but there was no difference in recovery for iADL and TUGWT. The percentage of patients who recovered at each time point was not different in both groups, and further study is needed to assess the clinical significance of these findings. However, this study provides high-quality prospective data on recovery patterns that will significantly benefit preoperative patient counseling. Exploratory analysis also revealed no difference in measures of independence between CUD and NCUD except for HGS. Scores showed a quicker return to baseline in patients undergoing CUD; however, larger studies are needed to validate these results. Compromised baseline scores did not affect 90-day complication rates or 1-year mortality in this analysis.

## Notes

Supplement 1.
Trial Protocol

Supplement 2. **eFigure 1.** ADL, iADL, TUGWT, and HGS Summary Scores of the Entire Cohort **eFigure 2.** ADL, iADL, TUGWT, and HGS Summary Scores by Surgical Approach (Study Groups) **eFigure 3.** ADL, iADL, TUGWT, and HGS Summary Scores by Type of Urinary Diversion **eFigure 4.** RAZOR Trial CONSORT Flow Diagram **eTable.** Recovery of HGS and ADL at 1 and 3 Months After Surgery

Supplement 3.
Data Sharing Statement
