# Supplementary material for: Comparison of Robot-Assisted and Open Radical Cystectomy in Recovery of Patient-Reported and Performance-Related Measures of Independence: A Secondary Analysis of a Randomized Clinical Trial
Source: JAMA Netw Open. 2022 Feb 16;5(2):e2148329. doi: 10.1001/jamanetworkopen.2021.48329 (PMC8851298; doi:10.1001/jamanetworkopen.2021.48329)
Supplement: Supplement 1. — Trial Protocol [file jamanetwopen-e2148329-s001.pdf]

Supplementary Appendix

This appendix has been provided by the authors to give readers additional information about their work. This supplement contains the following items:

1. Original protocol, final protocol, summary of changes.
2. The statistical plan is a part of the study protocols. The only change made in the statistical plan was to increase the subject accrual from 320 to 350 patients to ensure sufficient evaluable patients (n=288) for adequately powered statistical analyses.

The majority of changes made were procedural changes for clarification purposes.

Supplement to: **A Randomized trial of Robotic Versus Open Surgery for Bladder Cancer**

Dipen J. Parekh, Isildinha M. Reis, Erik P. Castle, Mark A. Gonzalgo, Michael E. Woods, Robert S. Svatek, Alon Z. Weizer, Badrinath R. Konety, Mathew Tollefson, Tracey L. Krupski, Norm D. Smith, Ahmed Shabsigh, Daniel A. Barocas, Marcus L Quek, Atreya Dash, Adam S. Kibel, Lynn Shemanski, Raj S. Pruthi, Jeff Montgomery, Christopher Weight, David S Sharp, Sam S. Chang, Michael S. Cookson, Gopal N. Gupta, Alex Gorbonos, Edward Uchio, Eila Skinner, Vivek Venkatramani, Nachiketh Soodana-Prakash, Kerri Kendrick, Joseph A Smith Jr., Ian M. Thompson

Table of Contents

1. Original protocol (v3/2/11) 3

2. Final protocol (v3/6/14) 40

3. Summary of changes 81

Original protocol – v3/2/11

Open Vs Robotic-Assisted Radical Cystectomy: A Randomized Trial

Protocol Number: 36911

Study Principal Investigator

Dipen J. Parekh, MD

Chief, Division of Urologic Oncology,

Director, Robotic Surgery, Associate Professor,

Department of Urology,

UTHSCSA

<u>Local Research Sites</u>:

The University of Texas Health Science Center at San Antonio

Christus Santa Rosa Hospital – Medical Center

Doctor’s Hospital at Renaissance (Edinburg, TX)

<u>Off-site Research Sites:</u>

Vanderbilt University Medical Center

Mayo Clinic, AZ

University of California at Irvine

Stanford University

Washington University

University of Minnesota

University of Virginia

University of North Carolina at Chapel Hill

The Ohio State University

University of Chicago

Regents of the University of Michigan

Loyola University

Data Management and Biostatistics:

Cancer Research and Biostatics (CRAB), Seattle, WA

Table of Contents

1. Responsible Entities and Staff 7
2. Background and Significance 11
3. Objectives and Specific Aims 14
4. Experimental Design 16
    - Inclusion/Exclusion criteria 16
    - Recruitment and consent procedures 17
    - Research Procedures 17
    - Clinical Procedures 22
    - Data Submission Procedures 25
5. Statistical Considerations 28
6. Human Subjects 33
    - Compensation 33
    - Risks to subject 33
    - Special precautions 33
    - Subject Completion and Withdrawal 35
    - Alternative treatments 35
    - Confidentiality 35
    - Data Safety Monitoring Committee 36
7. Literature Cited 37

APPENDIX A-ECOG Performance Status Scale 39

1. RESPONSIBLE ENTITIES AND STAFF

Principal Investigator: Dipen J. Parekh, MD

Associate Professor

The University of Texas Health Science Center at San Antonio

Co-Investigators:

Daniel A Barocas, MD

Assistant Professor

Vanderbilt University Medical Center

Erik Castle, MD

Associate Professor

Mayo Clinic, Scottsdale, AZ

John Crowley, PhD

President/CEO

Cancer Research And Biostatistics, Seattle, WA

Atreya Dash, MD

Assistant Professor

University of California at Irvine

Mark Gonzalgo, MD, PhD
Associate Professor
Stanford University

Adam Kibel, MD
Professor
Washington University

Badrinath Konety, MD
Professor
University of Minnesota

Tracey Krupski, MD
Assistant Professor
University of Virginia

Raj Pruthi, MD
Associate Professor
University of North Carolina at Chapel Hill

Ahmad Shabsigh, MD

Assistant Professor
The Ohio State University

Norm Smith, MD
Associate Professor
University of Chicago

Robert Svatek, MD
Assistant Professor
The University of Texas Health Science Center at San Antonio

Alon Weizer, MD
Assistant Professor
Regents of the University of Michigan

Michael Woods, MD
Assistant Professor
Loyola University

Consultants: Ian M Thompson, MD

Professor and Chairman,
Department of Urology
The University of Texas Health Science Center at San Antonio.

Valerie Lawrence, MD
Professor
Internal Medicine
The University of Texas Health Science Center at San Antonio.

Joseph A Smith Jr, MD
Professor and Chairman,
Department of Urology,
Vanderbilt University Medical Center

2. BACKGROUND AND SIGNIFICANCE

In 2008, approximately 69,000 patients were diagnosed with urinary bladder cancer and 14,000 patients were expected to die from bladder cancer [1]. Radical cystectomy with pelvic lymphadenectomy and urinary diversion is the standard of care for high grade carcinoma *in situ*, high grade recurrent superficial bladder cancer, or tumor invading the muscularis propria. Radical cystectomy with pelvic lymphadenectomy is associated with adequate survival outcomes and compares favorably with other treatment modalities [2,3]. However, this operation is an extremely complex and involved surgical procedure associated with considerable postoperative morbidity [4-6].

Traditionally, an incision is made from just above or at the level of umbilicus to the pubic symphysis. The bladder, prostate gland, and surrounding lymph nodes are removed followed by urinary diversion which consists of connecting the urinary tract into a segment of intestine[7]. The reported major and minor complication rates after open radical cystectomy are approximately 13% and 67% [4,5]. More recently, less invasive surgical treatments with robot-assisted laparoscopy have been advocated. Robot Assisted Radical Cystectomy (RARC) is one such approach where the radical cystectomy and pelvic lymph node dissection are accomplished by a robot assisted laparoscopic approach [8]. After the completion of the cystectomy and the lymph node dissection, a small periumbilical or infra-umbilical midline incision is made to complete the urinary diversion.

In other surgical procedures, such as nephrectomy, minimally invasive approaches have been

associated with less blood loss, shorter hospital stay, less use of pain medication and faster return to preoperative levels of independence[9, 10]. One would expect that RARC might offer similar advantages over open cystectomy. Furthermore, there is evidence that efforts to compensate for the insensible losses and blood loss associated with open surgery may contribute to the high complication rate[5]. Therefore, there is reason to expect that RARC might reduce complication rates compared to open cystectomy.

However, RARC is being marketed as superior to the traditional open operation without systematic comparative evaluations through randomized trials. The purported advantages of RARC from small-volume, single-institution series are decreased blood loss, decreased blood transfusion rates, decreased pain and opioid requirement, earlier time to oral intake, decreased hospital stay, fewer wound complications, and expedited perioperative and postoperative convalescence and recovery [11-13]. Additionally, there does not appear to be any compromise in the oncologic outcome as determined by pathology of surgical margins and number of pelvic lymph nodes removed [11-13]. However, despite these advantages, little is known regarding recovery to preoperative functional independence with RARC. Also, RARC is associated with a steep learning curve as well as significant cost enhancements. All outcome studies of RARC are either case series reports or comparative studies with major methodological limitations. Additionally, all are limited by major selection biases such as patients with more favorable oncologic, demographic, and clinical characteristics undergoing the RARC compared to the open approach [11,13]. Thus the perception that patient having RARC may have a better recovery is potentially due to the bias of preferential patient selection. Lastly, comparisons of open and

RARC should be cautiously interpreted since they have been performed by a variety of surgeons with different training and experience levels.

To date, no study has evaluated the outcomes measuring postoperative recovery using objective parameters of functional independence in patients undergoing RARC nor specifically compared it to outcomes following open cystectomy. Studies evaluating short-term Quality-of-Life (QOL) outcome measures following open cystectomy have not been performed yet. Lawrence et al. conducted a prospective cohort study to systematically describe the clinical course and predictors of long-term recovery to preoperative levels of functional independence in patients after major elective abdominal operations, using both self-report and performance-based instrument[14]. They found that a number of clinical factors independently predicted optimal functional recovery. Serious postoperative complications were consistent independent predictors of poorer recovery and longer time to recovery. Poor preoperative physical performance status (ECOG scale), serum creatinine > 1.5 mg/dL and albumin < 3 mg/dL independently predicted poor recovery at 6 months. However, to our knowledge, no one has tested the hypothesis that utilization of the robotic technique would independently improve recovery and result in superior short or long-term QOL outcomes.

3. OBJECTIVES AND SPECIFIC AIMS

Primary End Points

Specific Aim 1:

a. 1. Two-year progression free survival. *3-year progression free survival in 65% of patients.*

Specific Aim 2:

1. Serum Hemoglobin, Serum Creatinine and Serum Albumin levels at baseline and in the post-operative period at 4-6 weeks, 3 months, 6 months, 12 months, and 24 months.
2. Quality of Life (QOL) outcomes at baseline and in the post-operative period at 3 months and 6 months using the Functional Assessment of Cancer Therapy - Vanderbilt Cystectomy Index (FACT-VCI) as well as the Short Form 8 (SF-8) Questionnaire.
3. Compare surrogates of surgical quality by evaluating surgical margin status and number of lymph nodes harvested.
4. Compare surgical morbidity by evaluating complication rates at 90-days post-operative using the modified Clavien grading system.
5. Perioperative measures such as Estimated Blood Loss (EBL), Blood transfusion rates, total intraoperative fluid requirements, total operative time, total postoperative length of hospital stay and analgesic requirement.

Specific Aim 3:

A. Patient Reported Measures of Functional Independence

1. Activities of Daily Living (ADL) scores at baseline and in the post-operative period at 4-6 weeks, 3 months, and 6 months.
2. Instrumental Activities of Daily Living (IADL) scores at baseline and in the post-operative period at 4-6 weeks, 3 months, and 6 months.

B. Performance Related Measures of Functional Independence

1. Hand Grip Strength Test outcomes at baseline and in the post-operative period 4-6 weeks, 3 months, and 6 months.
2. Timed Up and Go Walking Test outcomes at baseline and in the post-operative period 4-6 weeks, 3 months, and 6 months.

Secondary End Points:

1. Compare fixed and variable costs associated with RARC and ORC operating room and hospital component.

4. EXPERIMENTAL DESIGN

This multi-institutional, randomized trial will enroll approximately 320 participants with approximately 160 participants in each arm of the trial at approximately 13 participating institutions. This study aims to determine whether Robotic-Assisted Radical Cystectomy (RARC) for treatment of bladder cancer provides a non-inferior oncologic control compared to traditional Open Radical Cystectomy (ORC), as measured by two-year progression-free survival. We propose a multi-institutional approach where participants randomized to both groups will have their surgery performed by experienced surgeons to eliminate institutional and surgeon bias.

Inclusion/Exclusion Criteria

Subject Inclusion Criteria:

1. Patient must have biopsy proven bladder cancer. Official pathology report reviewed at the participating institution is required.
2. Bladder cancer must be clinical stage T1-T4, N0-1, M0. (AJCC 7th edition)

Subject Exclusion Criteria:

1. Inability to give informed consent.
2. Prior major abdominal and pelvic open surgical procedures that would preclude a safe robotic approach, as determined by the treating surgeon.
3. At the discretion of the treating surgeon, any pre-existing condition such as severe chronic obstructive pulmonary disease that precludes a safe initiation or maintenance of pneumoperitoneum over a prolonged period of time and during surgery.

4. Age <18 or >99 years.
5. Pregnancy.

Recruitment and Consent Procedures

Patients who meet the eligibility criteria will be approached by research staff to determine whether they are willing to participate in the study. To eliminate selection bias, any patient who is determined to be a candidate for RARC will be given an option to participate in the study. The inclusion of patients eligible for this study will in no way compromise the quality of health care they will receive. Prior to study entry, the study staff will explain to each potential subject the research objectives, risks and benefits of study participation, alternative treatments available, and the subjects' rights and responsibilities. If the patient agrees to participate, informed consent will be obtained and, after consenting, randomization will take place.

Research Procedures

Randomization

Eligible, consented patients must be enrolled (i.e., randomized) no more than 60 days prior to surgery. This study uses a web-based patient enrollment and randomization system, through the data management services of Cancer Research And Biostatistics (CRAB). The patient enrollment/randomization eCRF (electronic Case Report Form) is accessed online through the study website at https://prodq.crab.org/Parekh/Login.aspx . Access is protected and available to authorized

users only. To request access to this system, or for questions or assistance using the website, please contact: WebhelpCRS@crab.org – please specify "Parekh Robotic Surgery" in the email subject line.

Treatment/Intervention Plan

Patients taking part in this study will be randomized using a dynamic balancing algorithm on type of diversion, within each institution as a block. Surgeons performing RARC and/or open radical cystectomy must have performed a minimum total of 10 over the past one year. Surgery must take place within 60 days of randomization.

The surgical approach, robotic versus open, is determined by randomization. All urinary diversions will be done via an open incision and the mode of diversion, whether intracorporeal or extracorporeal (orthotopic neobladder, continent cutaneous diversion or ileal conduit) will be selected by mutual agreement of the surgeon and patient, as is customarily done. The extent of the lymph node dissection will be determined by the surgeon but at minimum will include the external iliac, obturator, and hypogastric regions.

The following surgical templates will be implemented and adherence to these templates will be assessed by submission of the Surgeon's Intra-Op Data Form for all cases to CRAB:

Nodal templates (equivalent for robotic and open procedures)

Minimum LND (men and women) – all potential lymph node bearing tissue with the lateral limit

the genitofemoral nerve, distally Cooper's ligament to include the lymph node of Cloquet, proximally the crossing of the ureter over the common iliac vessels, medially the bladder to include the tissue medial to the hypogastric artery, posteriorly the floor of the obturator fossa with circumferential mobilization of the external iliac artery and vein.

Submission and processing of specimens

The cystectomy specimen (with or without uterus, ovaries, or vaginal cuff in females and prostate in males) will be submitted en bloc, processed and assessed in a standardized fashion at all the participating institutions for margin status along with histology, size, stage, grade and presence/absence of lymphovascular invasion. At a minimum, the LND will be submitted in two separate packets labeled left and right pelvic. All of these regions may be submitted in smaller packets (e.g. external iliac, obturator, internal iliac) at the surgeons' preference. The standardized Cystectomy Pathology Form will be submitted to CRAB.

The perioperative care measures will be performed per institutional standard based on each institution's policy.

Progression free survival (expressed in months) will be measured as the date of first recurrence minus the date of surgery for those who experience recurrence and as the total time on study for those who do not experience recurrence. Progression will be determined using RECIST 1.1 criteria by the treating physician based on radiographic or pathologic evidence of disease progression, or death from disease. Patients who survive beyond two years without evidence of progression will be censored at least once a year from the date of surgery until the conclusion of the trial. All patients will have been followed for at least 2 years and 65% of patients will

have been followed for 3 years. Patients who die of other causes within two years will be censored at the time of death. Patients lost to follow up will be censored at the date of last visit.

Overall survival (expressed in months) will be measured as the date of death minus the date of surgery for those who die while on study and as the total time on study for those who do not die while on study.

Serum hemoglobin and a comprehensive metabolic panel (CMP) will be measured on 10 cc of blood obtained by a venipuncture. These laboratory parameters are part of a routine preoperative work up and postoperative follow up in patients undergoing radical cystectomy and urinary diversion. No extra laboratory tests will be administered to the subjects enrolled in this trial.

Pathologic data will be obtained from the pathology reports after surgery with particular emphasis on the involvement of surgical margins with cancer, total number of lymph nodes harvested and their involvement with cancer as well as the pathologic stage of the tumor. All institutions will adopt a standardized procedure to process the cystectomy specimens along with the lymph nodes. A standardized form will be used to collect all the information pertaining to specimen processing and staging by the participating institutions. A copy of the pathologic form will be available in the patients' clinical records.

Perioperative mortality and morbidity will be evaluated using the modified Clavien grading system for complications by prospectively recording the intraoperative and post-operative complications until discharge and by patient interview during post discharge period until 4-6 weeks after surgery. The above data will be separately recorded in the patient's clinical records in an inpatient and outpatient setting.

Perioperative measures such as estimated blood loss (EBL), blood transfusion rates, total intraoperative fluid requirements, total operative time, total postoperative length of hospital stay and analgesic requirement will be prospectively recorded during the surgery and the postoperative hospital stay using the anesthesia, operative, nursing and inpatient medical records by a research coordinator. All medications will be converted to morphine equivalents (mEQ) by using the online calculator The Clinician's Ultimate Reference found at http://www.globalrph.com/narcoticonv.htm

Costs

We will obtain fixed and variable operating room costs by assessing amortized cost of robotic machine per case, amortized cost of maintenance per case, costs of dispensable equipment, cost of OR personnel and anesthesia resources per time. We will also obtain fixed and variable hospital costs based on length of stay. The above cost data will be collected from each participating center and data will be stored and analyzed by CRAB. We hypothesize that costs associated with robotic surgery will be no more than 5% of the costs associated with ORC.

Assessment	Baseline (Preoperativ	Hospital Discharg	4-6 Weeks	3 month	6 month	12 month	24 Months	36 Months

Only research personnel who are approved by the IRBs of the participating institutions will have access to study research information. All participating investigators are required to undergo and maintain CITI training.

Clinical Procedures

Measurements of Study Endpoints

Measurements of the study end points will be conducted according to the following Table 1:

Table 1: Study Calendar

	e)	e		s	s	s		
Baseline History and Physical Exam, Consent, Screening	√							
Randomization	√ (within 60 days prior to surgery)							
Progression Free Survival						√	√	√
Overall Survival			√	√	√	√	√	√
Activities of Daily Living (ADL) score	√		√	√	√			
Instrumental Activities of Daily Living (IADL) score	√		√	√	√			
Hand Grip Strength Test	√		√	√	√			
Timed Up and Go Walking Test	√		√	√	√			

Hemoglobin, BMP, serum albumin	√		√	√	√	√	√	√
Quality of Life Questionnaire (QOL) - Vanderbilt Cystectomy Index and SF8	√			√	√			
Obtain Pathology Reports for Surgical Margin Status and Lymph Node Count			√					
Surgical Complications per Modified Clavien Classification (AEs)/ Serious Adverse Events			√	√	√	√	√	√
Postoperative Complication Rates				√				
Imaging (CT scan /MRI of	√					√	√	√

Abdomen/Pelvis/C<br>hest)								
OR Costs<br>Hospital Costs		√						

Data Submission Procedures

Data must be submitted according to the following schedule:

- To perform randomization and obtain subject ID number: *Randomization Form.*

- Within 1 week following enrollment: All of the following forms:
    - *Medical History Form*
    - *Surgical History Form*
    - *Findings at TURBT Form*
    - *Hemoglobin, BMP, and Serum Albumin Form*
    - *Screening Physical Exam and Vital Signs Form*
    - *Baseline Disease Assessment Form*
    - *Baseline Vanderbilt Cystectomy Index QOL Questionnaire*
    - *Baseline SF-8 QOL Questionnaire*
    - *Baseline Activities of Daily Living QOL Questionnaire*
    - *Baseline Activities of Instrumental Activities of Daily Living QOL Questionnaire*
    - *Baseline Hand Grip Strength Form*

- *Baseline Timed Up and Go Walking Test Form*

- Within 1 week of each post-surgery laboratory assessment per Table 1**:**
    - *Hemoglobin, BMP, and Serum Albumin Form*

- Within 1 week following surgery: *Surgeon's Intra-Op Data Form.*

- Within 1 week following discharge for surgical hospitalization:
    - *Hospital Discharge Visit: OR and Hospital Costs Reporting Form*
    - *Surgeon's Post-Op Data Form*

- Within 1 week of each scheduled ADL, IADL, and QOL assessment per Table 1:
    - *Vanderbilt Cystectomy Index QOL Questionnaire*
    - *SF-8 QOL Questionnaire*
    - *Activities of Daily Living QOL Questionnaire*
    - *Activities of Instrumental Activities of Daily Living QOL Questionnaire*
    - *Hand Grip Strength Form*
    - *Timed Up and Go Walking Test Form*

- Within 2 weeks following each scheduled disease assessment/imaging exam per Table 1: *Post-Surgical Disease Assessment Form.*

- Within 4-6-weeks post-op: *Cystectomy Pathology Form*
- Within 1 week following 90-days post-op: *Surgeon's 90-Day Data Form*
- Within 1 week following 6-, 12-, 24-, and 36-months post-op (respectively):
  - *Post-Surgical Disease Assessment*
  - *Hematology Form*
  - *Serum Chemistry Form*
- Within 1week following each scheduled adverse events evaluation (per Table 1) until adverse events have resolved. Adverse event information is collected on the *Surgical Complications-Adverse Events Form.*
- Within the time frame and per the guidelines specified in section 5: *Serious Adverse Events*. Report per the instructions provided in section 5 AND flag as "SAE" on the *Surgical Complications-Adverse Events Form.*
- Within 2 weeks following knowledge of death, if death occurs prior to end of study: *Death Report Form*.

5. STATISTICAL CONSIDERATIONS

Study Objective and Primary Endpoint

The primary objective of this study is to compare progression-free survival of RARC versus ORC in patients with bladder cancer. More specifically, the primary endpoint for this study is progression-free survival at 2 years. This is a non-inferiority comparison, i.e. the study will test whether the robotic-assisted cystectomy is, at worst, inferior to the open radical cystectomy by a small pre-defined margin. This study will use a centralized dynamic allocation procedure to allocate an equal number of patients to each of the treatment arms. The procedure will balance the marginal distribution of the stratification factors between these two treatments.

Power and Significance

The margin for this study is 15% which means that RARC would be considered inferior if the true progression free survival at two years was more than 15% lower than the progression-free survival at two years in the ORC arm. A total of 288 evaluable patients (144 patients per arm) yield a study with 80% power and a significance level, alpha, of 5% to correctly reject the null-hypothesis of unacceptable inferiority. These calculations are based on the assumption that progression-free survival at two years in the patients receiving ORC is approximately 71% and that the rate of progressions at two years is binomially distributed[2, 17].

Stratification Factors

Because outcomes may vary by type of urinary diversion (ideal conduit or neobladder), and

ECOG Performance Status (0-1 vs. 2 or greater). We will also stratify by the clinical T stage of condition (T1, T2, T3, T4) since it directly influences oncologic outcomes.

Accrual and Study Duration

We anticipate participation of 13 sites and an accrual of approximately 110 eligible patients per year. Assuming a maximum drop-out rate of 10%, a total of 320 patients (160 patients in each arm) will be accrued to this study. Thus 320 patients will be accrued in approximately three years. All patients will be followed for at least two years for progression. Based on the accrual estimates, 65% of patients will have follow up available to evaluate progression free survival at 3 years. Thus, the study duration is expected to be approximately five years.

Secondary Endpoints

We will determine whether RARC is superior to ORC in terms of blood loss. More specifically, the overall blood loss due to surgery will be compared between the two treatment arms. In the ORC group the average blood loss is 575 ml [16]. 288 patients yield 90% power to detect a difference of blood loss between the two treatment groups of 20%. These calculations are based on the assumption that the amount of blood loss is normally distributed, that the average blood loss in the open surgery group is 575ml, and that the standard deviation of blood loss is 300ml. A one-sided significance level of 0.025 was used.

We will determine whether RARC is superior to ORC in terms of transfusion rates. The transfusion rates (proportion of patients requiring blood transfusions) will be compared

between the two treatment arms. The transfusion rate for ORC is approximately 75%(from Table 2, unpublished). 288 patients yield 92% power and a one-sided significance level of 0.025 to detect a difference of transfusion rates between arms of at least 20%. These calculations are based on the assumption that the transfusion rate is binomially distributed.

Length of hospital stay will be used as a surrogate for recovery after surgery. We will determine whether RARC is superior to ORC in terms of length of hospital stay. Currently, all of the patients receiving ORC stay in the hospital for more than 5 days while 67% of patients in the RARC group stay in the hospital for more than 5 days (from Table 2, unpublished). 288 patients yield 97% power and a one-sided significance level of 0.025 to detect a difference in percent of patients requiring a hospital stay beyond 5 days between RARC and ORC arms of at least 20%. These calculations are based on the assumption that the percent patients requiring a hospital stay beyond 5 days is binomially distributed.

Table 2 (Preliminary Data for Cost Analyses from University of North Carolina)

	ORC (n=21)	RARC (n=20)	P VALUE
Age (median)	70	70	
OR time (mins)	293	389	<0.001
OR fees (dollars)	5441	6202	<0.00001
OR disposables (dollars)	2485	3715	= 0.0003
OR capital + reusables	50	2000	
LOS (days)	6	4	0.02 (Mann-Whitney U)

Room and Board	5954	3664	0.005 (Mann-Whitney U)
Overall Costs	19047	19837	0.14 (Mann-Whitney U)

Hand Grip Strength at 3 months after surgery will be measured as a surrogate for recovery after surgery. It was found that only 39% of patients recovered at three months after a major abdominal surgery as measured by the Hand Grip Strength[14].We will compare the proportion of patients recovered as measured by Hand Grip Strength between the two treatment arms. We hypothesize that 20% more patients will have recovered three months after surgery in the RARC arm compared to the ORC arm. A total of 288 patients yield 91% power and a one-sided significance level of 0.025 to detect a difference between arms of at least 20%. These calculations are based on the assumption that Hand Grip Strength at three months is binomially distributed.

Surgical margin as a measure for local cancer control will be measured as positive or negative for each patient and compared between arms using a Fisher's exact test. The number of nodes resected in each arm will be compared using a t-test.

Overall survival will be evaluated using the method of Kaplan Meyer and comparisons between arms will be made using the log-rank test.

6. HUMAN SUBJECTS

Compensation

There will be no compensation provided to subjects for participating in the study.

Risks to Subjects

The only research related risks to subjects are the potential loss of PHI and potential mental distress during conduct of questionnaires. Measures to maintain confidentiality are being employed. For a detailed description, please see the section titled "Confidentiality." Should patients express discomfort during questionnaires or other assessments, they will first be allowed to take a break from questioning. If distress persists, the session will be terminated. Participants may refuse to answer questions which cause them discomfort rather than being withdrawn from the study.

Special Precautions

Subject data will be examined at each follow up visit and subjects queried for adverse events (AE) defined as complications related to the robotic/open cystectomy and/or study procedures. An AE or complication is the appearance of undesirable sign(s), symptom(s), or medical condition(s) occurring after a participant signs the informed consent and considered to be related to the robotic/open cystectomy and/or study procedures. A serious adverse event (SAE) is any untoward medical occurrence that:

1. Is fatal or life-threatening
2. Requires hospitalization or prolongation of existing hospitalization
3. Results in disability/incapacity
4. Is medically significant in that it may jeopardize the patient and may require medical or surgical intervention to prevent one of the outcomes listed above.

All AEs or complications will be graded for severity according to the modified Clavien grading system (Appendix E). All adverse events will be reported to the IRB at the time of annual review and to the DSMC as described below.

The investigator is obligated to assess the relationship between any study-related procedure and the occurrence of each SAE. The investigator will use clinical judgment to determine the relationship. Alternative causes, such as natural history of the underlying diseases, concomitant therapy, other risk factors, and the temporal relationship of the event to any study-related procedure will be considered and investigated.

Even in situations when an SAE has occurred and the investigator has incomplete information to include in the initial SAE report, the investigator will make an assessment of causality for every event prior to reporting it. The investigator may change his opinion of causality in light of follow-up information and amend the SAE case report form and report accordingly.

SAEs meeting the IRB definition of Unanticipated Problems Involving Risk to Subjects or others (UPIRSO) will be reported to the IRB within 7 days, and within 48 hours if life-threatening or fatal. All SAEs will also be reported to the coordinating center office of Dr Parekh at the University of Texas Health Sciences Center at San Antonio. After completion of review by Dr

Parekh, SAEs will be summarized and communicated across sites via the posting to the study website at https://prodq.crab.org/Parekh/Login.aspx.

The data will be reviewed on a biweekly basis by the investigators to ensure quality control and safety. During the study when there is a safety evaluation, the investigator and/or research staff will be responsible for detecting, documenting and reporting any adverse events or serious adverse events to the IRB.

Subject Completion and Withdrawal

A subject will be considered completed when he/she has completed all follow-up visits up to the 24-month evaluation. A subject may discontinue participation in this study at any time at the investigator's discretion or at the request of the subject. The reason for study withdrawal will be documented in the study related source documentation.

Alternative Treatments

Subjects who are eligible for the proposed study will be randomized to receive either open or robotic-assisted cystectomy. The alternative to participating in this study would be for a subject to choose which surgical technique will be used rather than being randomized to one or the other.

Confidentiality

Maintaining confidentiality of patient-specific information will be top priority throughout all phases of the study. Patient data (PHI) will be compiled in a database and de-identified upon

completion of analysis. Cases in the database will be identified by initials, patient identification number, the year of patient's birth, and the date of surgery. The database will not include information which can be identifiable with the link (or key). All electronic data will be stored in a password protected database in accordance with institutional computer-security policies. The identification number is actually a research record number that cannot be linked to the subject except by a key. This key will be maintained by the Principal Investigator in room 306L of the UTHSCSA Medical School. This room is kept locked and access is limited. The data will be stored and maintained by the CRAB informatics core facility.

Data Safety Monitoring Committee

A Data and Safety Monitoring Board will oversee the conduct of the study. The Board consists of 3 voting, independent members: 1 surgeon, 1 statistician, and 1 lay person. Non-voting members include support staff from Cancer Research And Biostatistics (who will prepare the DSMC reports), and project faculty (Principal Investigators) as appropriate. DSMC members receive database summaries from CRAB, including adverse events and post-surgical complications reports, serious adverse event summaries, and other pertinent patient/treatment summary information. Meetings occur every six months, convened via teleconference. The DSMC is responsible for decisions regarding possible termination and/or early reporting of the study.

7. LITERATURE CITED

1. Jemal A, Siegel R, Ward E, et al. Cancer statistics, 2008. CA: a cancer journal for clinicians 2008;58(2):71-96.
2. Stein JP, Lieskovsky G, Cote R, et al. Radical cystectomy in the treatment of invasive bladder cancer: long-term results in 1,054 patients. J Clin Oncol 2001;19(3):666-75.
3. Stein JP, Skinner DG. Results with radical cystectomy for treating bladder cancer: a 'reference standard' for high-grade, invasive bladder cancer. BJU international 2003;92(1):12-7.
4. Cookson MS, Chang SS, Wells N, Parekh DJ, Smith JA, Jr. Complications of radical cystectomy for nonmuscle invasive disease: comparison with muscle invasive disease. The Journal of urology 2003;169(1):101-4.
5. Shabsigh A, Korets R, Vora KC, et al. Defining Early Morbidity of Radical Cystectomy for Patients with Bladder Cancer Using a Standardized Reporting Methodology. European urology 2008.
6. Konety BR, Allareddy V, Herr H. Complications after radical cystectomy: analysis of population-based data. Urology 2006;68(1):58-64.
7. Stein JP, Skinner DG. Surgical atlas. Radical cystectomy. BJU international 2004;94(1):197-221.
8. Menon M, Hemal AK, Tewari A, et al. Robot-assisted radical cystectomy and urinary diversion in female patients: technique with preservation of the uterus and vagina. Journal of the American College of Surgeons 2004;198(3):386-93.
9. Gill IS. Laparoscopic radical nephrectomy for cancer. The Urologic clinics of North America 2000;27(4):707-19.

10. Dillenburg W, Poulakis V, Skriapas K, et al. Retroperitoneoscopic versus open surgical radical nephrectomy for large renal cell carcinoma in clinical stage cT2 or cT3a: quality of life, pain and reconvalescence. European urology 2006;49(2):314-22; discussion 22-3.

11. Wang GJ, Barocas DA, Raman JD, Scherr DS. Robotic vs open radical cystectomy: prospective comparison of perioperative outcomes and pathological measures of early oncological efficacy. BJU international 2008;101(1):89-93.

12. Guru KA, Kim HL, Piacente PM, Mohler JL. Robot-assisted radical cystectomy and pelvic lymph node dissection: initial experience at Roswell Park Cancer Institute. Urology 2007;69(3):469-74.

13. Dasgupta P, Rimington P, Murphy D, Elhage O, Challacombe B, Khan MS. Robotically assisted radical cystectomy. BJU international 2008;101(12):1489-90.

14. Lawrence VA, Hazuda HP, Cornell JE, et al. Functional independence after major abdominal surgery in the elderly. Journal of the American College of Surgeons 2004;199(5):762-72.

15. Fitzmaurice GM LN, Ware JH. . Applied longitudinal analysis. NY. John Wiley & Sons 2004.

16. Probstfield JL, Wittes JT, Hunninghake DB. Recruitment in NHLBI population-based studies and randomized clinical trials: data analysis and survey results. Controlled clinical trials 1987;8(4 Suppl):141S-9S.

17. Moyé L. Statistical monitoring of clinical trials:. Fundamentals for investigators 2006.;NY: Springer.

Appendix A: ECOG [a] Performance Status Scale

Grade	Status
0	Fully active, able to carry on all pre-disease performance without restriction
1	Restricted in physically strenuous activity but ambulatory and able to carry out work of a light or sedentary nature, eg, light house work, office work
2	Ambulatory and capable of all selfcare but unable to carry out any work activities. Up and about more than 50% of waking hours
3	Capable of only limited selfcare, confined to bed or chair more than 50% of waking hours
4	Completely disabled. Cannot carry on any selfcare. Totally confined to bed or chair
5	Dead

[a] As published in *Am J Clin Oncol (CCT)*. 1982;5:649-655.

Final protocol – v3/6/14

Open Vs Robotic-Assisted Radical Cystectomy: A Randomized Trial

Protocol Number 36911

Study Principal Investigator

Dipen J. Parekh, MD

Professor and Chair,

Department of Urology,

University of Miami School of Medicine

<u>Research Sites:</u>

University of Miami

The University of Texas Health Science Center at San Antonio

Vanderbilt University Medical Center

Mayo Clinic, AZ

Mayo Clinic, MN

University of California at Irvine

Stanford University

Brigham and Women's Hospital

University of Minnesota

University of Virginia

University of North Carolina at Chapel Hill

The Ohio State University

University of Chicago

Regents of the University of Michigan

Loyola University

Data Management and Biostatistics:

Cancer Research and Biostatics (CRAB), Seattle, WA

Table of Contents

1. Responsible Entities and Staff 44
2. Background and Significance 49
3. Objectives and Specific Aims 52
4. Experimental Design 54
   Inclusion/Exclusion criteria 54
   Recruitment and consent procedures 55
   Research Procedures 55
   Clinical Procedures 60
   Data Submission Procedures 63
5. Statistical Considerations 67
6. Human Subjects 74
   Compensation 74
   Risks to subject 74
   Special precautions 74
   Subject Completion and Withdrawal 76
   Alternative treatments 76
   Confidentiality 76
   Data Safety Monitoring Committee 77
7. Literature Cited 78

APPENDIX A – ECOG Performance Status Scale 80

1. RESPONSIBLE ENTITIES AND STAFF

Principal Investigator: Dipen J. Parekh, MD

Professor and Chair,

University of Miami

Co-Investigators:

Daniel A Barocas, MD

Assistant Professor

Vanderbilt University Medical Center

Erik Castle, MD

Associate Professor

Mayo Clinic, Scottsdale, AZ

John Crowley, PhD

President/CEO

Cancer Research And Biostatistics, Seattle, WA

Atreya Dash, MD

Associate Professor

University of Washington

Mark Gonzalgo, MD, PhD
Associate Professor
University of Miami

Adam Kibel, MD
Chair of Urology/Professor
Brigham and Women’s Hospital

Badrinath Konety, MD
Professor
University of Minnesota

Tracey Krupski, MD
Assistant Professor
University of Virginia

Raj Pruthi, MD
Associate Professor
University of North Carolina at Chapel Hill

Ahmad Shabsigh, MD

Assistant Professor
The Ohio State University

Eila Skinner, MD
Chair of Urology/Professor
Stanford University

Norm Smith, MD
Associate Professor
University of Chicago

Robert Svatek, MD
Assistant Professor
The University of Texas Health Science Center at San Antonio

Matthew Tollefson, MD
Assistant Professor
Mayo Clinic, Rochester, MN

Edward Uchio, MD
Associate Professor
University of California at Irvine

Alon Weizer, MD
Assistant Professor
Regents of the University of Michigan

Michael Woods, MD
Associate Professor
University of North Carolina at Chapel Hill

Marcus Quek, MD
Associate Professor
Loyola University

Consultants: Ian M Thompson, MD
Professor
Department of Urology
The University of Texas Health Science Center at San Antonio.

Valerie Lawrence, MD
Professor

Internal Medicine
The University of Texas Health Science Center at San Antonio.

Joseph A Smith Jr, MD
Professor and Chairman,
Department of Urology,
Vanderbilt University Medical Center

2. BACKGROUND AND SIGNIFICANCE

In 2008, approximately 69,000 patients were diagnosed with urinary bladder cancer and 14,000 patients were expected to die from bladder cancer [1]. Radical cystectomy with pelvic lymphadenectomy and urinary diversion is the standard of care for high grade carcinoma *in situ*, high grade recurrent superficial bladder cancer, or tumor invading the muscularis propria. Radical cystectomy with pelvic lymphadenectomy is associated with adequate survival outcomes and compares favorably with other treatment modalities [2,3]. However, this operation is an extremely complex and involved surgical procedure associated with considerable postoperative morbidity [4-6].

Traditionally, an incision is made from just above or at the level of umbilicus to the pubic symphysis. The bladder, prostate gland, and surrounding lymph nodes are removed followed by urinary diversion which consists of connecting the urinary tract into a segment of intestine[7]. The reported major and minor complication rates after open radical cystectomy are approximately 13% and 67% [4,5]. More recently, less invasive surgical treatments with robot-assisted laparoscopy have been advocated. Robot Assisted Radical Cystectomy (RARC) is one such approach where the radical cystectomy and pelvic lymph node dissection are accomplished by a robot assisted laparoscopic approach [8]. After the completion of the cystectomy and the lymph node dissection, a small periumbilical or infra-umbilical midline incision is made to complete the urinary diversion.

In other surgical procedures, such as nephrectomy, minimally invasive approaches have been associated with less blood loss, shorter hospital stay, less use of pain medication and faster return to preoperative levels of independence[9, 10]. One would expect that RARC might offer similar advantages over open cystectomy. Furthermore, there is evidence that efforts to compensate for the insensible losses and blood loss associated with open surgery may contribute to the high complication rate[5]. Therefore, there is reason to expect that RARC might reduce complication rates compared to open cystectomy.

However, RARC is being marketed as superior to the traditional open operation without systematic comparative evaluations through randomized trials. The purported advantages of RARC from small-volume, single-institution series are decreased blood loss, decreased blood transfusion rates, decreased pain and opioid requirement, earlier time to oral intake, decreased hospital stay, fewer wound complications, and expedited perioperative and postoperative convalescence and recovery [11-13]. Additionally, there does not appear to be any compromise in the oncologic outcome as determined by pathology of surgical margins and number of pelvic lymph nodes removed [11-13]. However, despite these advantages, little is known regarding recovery to preoperative functional independence with RARC. Also, RARC is associated with a steep learning curve as well as significant cost enhancements. All outcome studies of RARC are either case series reports or comparative studies with major methodological limitations. Additionally, all are limited by major selection biases such as patients with more favorable oncologic, demographic, and clinical characteristics undergoing the RARC compared to the open approach [11,13]. Thus the perception that patient having RARC may have a better recovery is

potentially due to the bias of preferential patient selection. Lastly, comparisons of open and RARC should be cautiously interpreted since they have been performed by a variety of surgeons with different training and experience levels.

To date, no study has evaluated the outcomes measuring postoperative recovery using objective parameters of functional independence in patients undergoing RARC nor specifically compared it to outcomes following open cystectomy. Studies evaluating short-term Quality-of-Life (QOL) outcome measures following open cystectomy have not been performed yet. Lawrence et al. conducted a prospective cohort study to systematically describe the clinical course and predictors of long-term recovery to preoperative levels of functional independence in patients after major elective abdominal operations, using both self-report and performance-based instrument[14]. They found that a number of clinical factors independently predicted optimal functional recovery. Serious postoperative complications were consistent independent predictors of poorer recovery and longer time to recovery. Poor preoperative physical performance status (ECOG scale), serum creatinine > 1.5 mg/dL and albumin < 3 mg/dL independently predicted poor recovery at 6 months. However, to our knowledge, no one has tested the hypothesis that utilization of the robotic technique would independently improve recovery and result in superior short or long-term QOL outcomes.

3. OBJECTIVES AND SPECIFIC AIMS

Primary End Points

Specific Aim 1:

1. Two-year progression free survival.

Specific Aim 2:

1. Serum Hemoglobin, Serum Creatinine and Serum Albumin levels at baseline and in the post-operative period at 4-6 weeks, 3 months, 6 months, 12 months, 24 months, and 36 months.
2. Quality of Life (QOL) outcomes at baseline and in the post-operative period at 3 months and 6 months using the Functional Assessment of Cancer Therapy - Vanderbilt Cystectomy Index (FACT-VCI) as well as the Short Form 8 (SF-8) Questionnaires.
3. Compare surrogates of surgical quality by evaluating surgical margin status and number of lymph nodes harvested.
4. Compare surgical morbidity by evaluating complication rates at 90-days post-operative using the modified Clavien grading system.
5. Perioperative measures such as Estimated Blood Loss (EBL), Blood transfusion rates, total intraoperative fluid requirements, total operative time, total postoperative length of hospital stay and analgesic requirement.
6. *3-year progression free survival in 65% of patients.*

Specific Aim 3:

A. Patient Reported Measures of Functional Independence

1. Activities of Daily Living (ADL) scores at baseline and in the post-operative period at 4-6 weeks, 3 months, and 6 months.
2. Instrumental Activities of Daily Living (IADL) scores at baseline and in the post-operative period at 4-6 weeks, 3 months, and 6 months.

B. Performance Related Measures of Functional Independence

1. Hand Grip Strength Test outcomes at baseline and in the post-operative period 4-6 weeks, 3 months, and 6 months.
2. Timed Up and Go Walking Test outcomes at baseline and in the post-operative period 4-6 weeks, 3 months, and 6 months.

Secondary End Points:

1. Compare fixed and variable costs associated with RARC and ORC operating room and hospital component.

4. EXPERIMENTAL DESIGN

This multi-institutional, randomized trial will enroll approximately 350 participants with approximately 175 participants in each arm of the trial at approximately 15 participating institutions. This study aims to determine whether Robotic-Assisted Radical Cystectomy (RARC) for treatment of bladder cancer provides a non-inferior oncologic control compared to traditional Open Radical Cystectomy (ORC), as measured by two-year progression-free survival. We propose a multi-institutional approach where participants randomized to both groups will have their surgery performed by experienced surgeons to eliminate institutional and surgeon bias.

Inclusion/Exclusion Criteria

Subject Inclusion Criteria:

1. Patient must have biopsy proven bladder cancer. Official pathology report reviewed at the participating institution is required.
2. Bladder cancer must be clinical stage T1-T4, N0-1, M0. (AJCC 7th edition) or refractory cis (carcinoma in situ).

Subject Exclusion Criteria:

1. Inability to give informed consent.
2. Prior major abdominal and pelvic open surgical procedures that would preclude a safe robotic approach, as determined by the treating surgeon.

3. At the discretion of the treating surgeon, any pre-existing condition such as severe chronic obstructive pulmonary disease that precludes a safe initiation or maintenance of pneumoperitoneum over a prolonged period of time and during surgery.
4. Age <18 or >99 years.
5. Pregnancy.

Recruitment and Consent Procedures

Patients who meet the eligibility criteria will be approached by research staff to determine whether they are willing to participate in the study. To eliminate selection bias, any patient who is determined to be a candidate for RARC will be given an option to participate in the study. The inclusion of patients eligible for this study will in no way compromise the quality of health care they will receive. Prior to study entry, the study staff will explain to each potential subject the research objectives, risks and benefits of study participation, alternative treatments available, and the subjects' rights and responsibilities. If the patient agrees to participate, informed consent will be obtained and, after consenting, randomization will take place. Participation in this study will last up to approximately 5 years.

Research Procedures

Randomization

Eligible, consented patients must be enrolled (i.e., randomized) no more than 60 days prior to surgery. This study uses a web-based patient enrollment and randomization system, through the data management services of Cancer Research And

Biostatistics (CRAB). The patient enrollment/randomization eCRF (electronic Case Report Form) is accessed online through the study website at https://prodq.crab.org/Parekh/Login.aspx. Access is protected and available to authorized users only. To request access to this system, or for questions or assistance using the website, please contact: WebhelpCRS@crab.org – please specify "Parekh Robotic Surgery" in the email subject line.

Treatment/Intervention Plan:

Patients taking part in this study will be randomized using a dynamic balancing algorithm on type of diversion, within each institution as a block. Surgeons performing RARC and/or open radical cystectomy must have performed a minimum total of 10 over the past one year. Surgery must take place within 60 days of randomization.

The surgical approach, robotic versus open, is determined by randomization. All urinary diversions will be done via an open incision and the mode of diversion, whether intracorporeal or extracorporeal (orthotopic neobladder, continent cutaneous diversion or ileal conduit) will be selected by mutual agreement of the surgeon and patient, as is customarily done. The extent of the lymph node dissection will be determined by the surgeon but at minimum will include the external iliac, obturator, and hypogastric regions.

The following surgical templates will be implemented and adherence to these templates will be assessed by submission of the Surgeon's Intra-Op Data Form for all cases to CRAB:

Nodal templates (equivalent for robotic and open procedures)

Minimum LND (men and women) – all potential lymph node bearing tissue with the lateral limit the genitofemoral nerve, distally Cooper's ligament to include the lymph node of Cloquet, proximally the crossing of the ureter over the common iliac vessels, medially the bladder to include the tissue medial to the hypogastric artery, posteriorly the floor of the obturator fossa with circumferential mobilization of the external iliac artery and vein.

Submission and processing of specimens

The cystectomy specimen (with or without uterus, ovaries, or vaginal cuff in females and prostate in males) will be submitted en bloc, processed and assessed in a standardized fashion at all the participating institutions for margin status along with histology, size, stage, grade and presence/absence of lymphovascular invasion. At a minimum, the LND will be submitted in two separate packets labeled left and right pelvic. All of these regions may be submitted in smaller packets (e.g. external iliac, obturator, internal iliac) at the surgeons' preference. The standardized Cystectomy Pathology Form will be submitted to CRAB.

The perioperative care measures will be performed per institutional standard based on each institution's policy.

Progression free survival: From the date of surgery to the date of first documentation of progression or death due to any cause. Patients last known to be alive and progression-free are censored at the date of last contact. Progression will be determined using RECIST 1.1 criteria by

the treating physician based on radiographic or pathologic evidence of disease progression, or death from disease. Any documented recurrence will be considered progression. All patients will have been followed for at least 2 years and 65% of patients will have been followed for 3 years.

Overall survival: From date of surgery to date of death due to any cause. Patients last known to be alive are censored at the date of last contact.

Serum hemoglobin and a comprehensive metabolic panel (CMP) will be measured on 10 cc of blood obtained by a venipuncture. These laboratory parameters are part of a routine preoperative work up and postoperative follow up in patients undergoing radical cystectomy and urinary diversion. No extra laboratory tests will be administered to the subjects enrolled in this trial.

Pathologic data will be obtained from the pathology reports after surgery with particular emphasis on the involvement of surgical margins with cancer, total number of lymph nodes harvested and their involvement with cancer as well as the pathologic stage of the tumor. All institutions will adopt a standardized procedure to process the cystectomy specimens along with the lymph nodes. A standardized form will be used to collect all the information pertaining to specimen processing and staging by the participating institutions. A copy of the pathologic form will be available in the patients' clinical records.

Perioperative mortality and morbidity will be evaluated using the modified Clavien grading

system for complications by prospectively recording the intraoperative and post-operative complications until discharge and by patient interview during post discharge period until 4-6 weeks after surgery. The above data will be separately recorded in the patient's clinical records in an inpatient and outpatient setting.

Perioperative measures such as estimated blood loss (EBL), blood transfusion rates, total intraoperative fluid requirements, total operative time, total postoperative length of hospital stay and analgesic requirement will be prospectively recorded during the surgery and the postoperative hospital stay using the anesthesia, operative, nursing and inpatient medical records by a research coordinator. All medications will be converted to morphine equivalents by using the online calculator The Clinician's Ultimate Reference found at http://www.globalrph.com/narcoticonv.htm

Costs

We will obtain fixed and variable operating room costs by assessing amortized cost of robotic machine per case, amortized cost of maintenance per case, costs of dispensable equipment, cost of OR personnel and anesthesia resources per time. We will also obtain fixed and variable hospital costs based on length of stay. The above cost data will be collected from each participating center and data will be stored and analyzed by CRAB. We hypothesize that costs associated with robotic surgery will be no more than 5% of the costs associated with ORC. Only research personnel who are approved by the IRBs of the participating institutions will have access to study research information. All participating investigators are required to undergo

and maintain CITI training.

Clinical Procedures

Measurements of Study Endpoints

Measurements of the study end points will be conducted according to the following Table 1:

Table 1: Study Calendar

**Assessment**	**Baseline (Preoperative)**	**Hospital Discharge** (±2 weeks)	**4-6 Weeks**	**3 months**	**6 months**	**12 months**	**24 Months**	**36 Months**
Baseline History and Physical Exam, Consent, Screening, ECOG Performance Status, TURBT findings	√							
Randomization	√ (within 60 days prior to surgery)							
Progression Free Survival						√	√	√

Overall Survival			√	√	√	√	√	√
Activities of Daily Living (ADL) score	√		√ (±30 days)	√ (±30 days)	√ (±30 days)			
Instrumental Activities of Daily Living (IADL) score	√		√ (±30 days)	√ (±30 days)	√ (±30 days)			
Hand Grip Strength Test	√		√ (±30 days)	√ (±30 days)	√ (±30 days)			
Timed Up and Go Walking Test	√		√ (±30 days)	√ (±30 days)	√ (±30 days)			
Hemoglobin, BMP, serum albumin	√		√	√	√	√	√	√
Quality of Life Questionnaire (QOL) - Vanderbilt Cystectomy Index and SF8	√			√ (±30 days)	√ (±30 days)			

Obtain Pathology Reports for Surgical Margin Status and Lymph Node Count			√					
Surgical Complications per Modified Clavien Classification (AEs)/ Serious Adverse Events			√	√	√	√	√	√
Postoperative Complication Rates (Surgeon's 90-Day Data Form)				√				
Imaging (CT scan /MRI /Xray/etc of Abdomen/Pelvis/Chest/etc)	√					√	√	√
OR Costs Hospital Costs		√						
Surgeon's Intra Op		Post Op						

Data, Cystectomy Pathology								
Length of Hospital Stay, Analgesics, Complications (Surgeon's Post Op Data Form)		√						
Target Lesions (Post Surgical Disease Assessment Form) to document cancer progression, according to local site Standard of Care.					√	√	√	√

Data Submission Procedures

Data must be submitted according to the following schedule:

- <u>To perform randomization and obtain subject ID number</u>:
  Randomization Form.

- Within 1 week following enrollment: All of the following forms:
  - *Medical History Form*
  - *Surgical History Form*
  - *Findings at TURBT Form*
  - *Hemoglobin, BMP, and Serum Albumin Form*
  - *Screening Physical Exam and Vital Signs Form*
  - *Baseline Disease Assessment Form*
  - *Baseline Vanderbilt Cystectomy Index QOL Questionnaire*
  - *Baseline SF-8 QOL Questionnaire*
  - *Baseline Activities of Daily Living QOL Questionnaire*
  - *Baseline Activities of Instrumental Activities of Daily Living QOL Questionnaire*
  - *Baseline Hand Grip Strength Form*
  - *Baseline Timed Up and Go Walking Test Form*
- Within 1 week of each post-surgery laboratory assessment per Table 1**:**
  - *Hemoglobin, BMP, and Serum Albumin Form*
- Within 1 week following surgery: *Surgeon's Intra-Op Data Form.*
- Within 1 week following discharge for surgical hospitalization:
  - *Hospital Discharge Visit: OR and Hospital Costs Reporting Form*
  - *Surgeon's Post-Op Data Form*
- Within 1 week of each scheduled ADL, IADL, and QOL assessment per Table 1:
  - *Vanderbilt Cystectomy Index QOL Questionnaire*

- *SF-8 QOL Questionnaire*
- *Activities of Daily Living QOL Questionnaire*
- *Activities of Instrumental Activities of Daily Living QOL Questionnaire*
- *Hand Grip Strength Form*
- *Timed Up and Go Walking Test Form*

- Within 2 weeks following each scheduled disease assessment/imaging exam per Table 1: *Post-Surgical Disease Assessment Form.*
- Within 4-6-weeks post-op: *Cystectomy Pathology Form*
- Within 1 week following 90-days post-op: *Surgeon's 90-Day Data Form*
- Within 1 week following 6-, 12-, 24-, and 36-months post-op (respectively):
    - *Post-Surgical Disease Assessment*
    - *Hematology Form*
    - *Serum Chemistry Form*
- Within 1week following each scheduled adverse events evaluation (per Table 1) until adverse events have resolved. Adverse event information is collected on the *Surgical Complications-Adverse Events Form.*
- Within the time frame and per the guidelines specified in section 6: *Serious Adverse Events*. Report per the instructions provided in section 6 AND flag as "SAE" on the *Surgical Complications-Adverse Events*

Form.

- Within 2 weeks following knowledge of death, if death occurs prior to end of study: *Death Report Form*.

5. STATISTICAL CONSIDERATIONS

Study Objective and Primary Endpoint

The primary objective of this study is to compare progression-free survival of RARC versus ORC in patients with bladder cancer. More specifically, the primary endpoint for this study is progression-free survival at 2 years. This is a non-inferiority comparison, i.e. the study will test whether the robotic-assisted cystectomy is, at worst, inferior to the open radical cystectomy by a small pre-defined margin. This study will use a centralized dynamic allocation procedure to allocate an equal number of patients to each of the treatment arms. The procedure will balance the marginal distribution of the stratification factors between these two treatments.

Power and Significance

The margin for this study is 15% which means that RARC would be considered inferior if the true progression free survival at two years was more than 15% lower than the progression-free survival at two years in the ORC arm. A total of 288 evaluable patients (144 patients per arm) yield a study with 80% power and a two-sided significance level, alpha, of 5% to correctly reject the null-hypothesis of unacceptable inferiority. These calculations are based on the assumption that progression-free survival at two years in the patients receiving ORC is approximately 71% and that the rate of progressions at two years is binomially distributed[2, 17]. Evaluable patients are defined as eligible patients who have no major protocol deviations and have 90-day post-surgery follow-up data. Major protocol deviations will be recorded for patients with no surgery given, where surgery was started correctly but discontinued before cystectomy was completed;

or where the patient received a surgery type different from their randomization assignment for any reason. In addition, a major protocol deviation will be recorded if the surgery begins as assigned, but the robotic procedure is aborted and an open procedure is required to complete the operation.

Stratification Factors

Because outcomes may vary by type of urinary diversion (ideal conduit or neobladder), we will also stratify by the clinical T stage of condition (T1, T2, T3, T4) and neo adjuvant chemotherapy since it directly influences oncologic outcomes.

Accrual and Study Duration

We anticipate participation of 15 sites and an accrual of approximately 110 eligible patients per year. Assuming a maximum drop-out rate of 10%, a total of approximately 350 patients (approximately 175 patients in each arm) will be accrued to this study. Thus approximately 350 patients will be accrued in approximately three years. All patients will be followed for at least two years for progression. Based on the accrual estimates, after three years of accrual and two additional years of follow-up, 65% of patients will have follow up available to evaluate progression free survival at 3 years. Thus, the study duration is expected to be approximately five years.

Analysis of Primary Endpoint

A one-sided Mantel-Haenszel test with half the alpha (0.025) will be used for testing the primary non-inferiority hypothesis that compares progression free survival at two years in the two treatment arms.

In superiority trials the intent to treat (ITT) population is widely accepted as the analysis population for the primary endpoint as it gives the most conservative result of such a study. In contrast, for non-inferiority trials the inclusion of ineligible or untreated patients or the lack of adherence to the assigned treatment is expected to increase the noise of the study and make the two treatment arms look more alike, thus the overall results of the study less conservative. Thus, we will use the per-protocol (PP) population as the analysis population for the primary endpoint. We will also perform a sensitivity analysis of the primary endpoint using the ITT population. These two analysis populations are defined below. The design and analysis of this trial are based on the SWOG standards for non-inferiority trials, which are detailed the chapter 12 in the Handbook for Statistics in Clinical Oncology, second edition, Chapman & Hall 2006.

Serum hemoglobin, serum creatinine and serum albumin levels will be taken at baseline and at a variety of time points post-surgery throughout the study. Linear mixed effects will be used to compare these blood levels and their changes over time between the two treatment groups.

QOL outcomes will be measured at baseline and in the post-operative period at 3 and 6 months using the Functional Assessment of Cancer Therapy – Vanderbilt Cystectomy Index (FACT-VCI) as well as the Short Form 8 (SF-8) Questionnaires. Simple descriptive statistics, such as mean

and standard deviation, will be used to summarize the FACT-VCI and the SF-8 scores at each time point and for each treatment group. A multivariate linear mixed effects model will then be fitted to each score in this repeated measures design. The main effect will be visit (at baseline, 3 and 6 months) and will be treated as a categorical variable to accommodate for the non-linear trends. If the exact time corresponding to a particular visit differs significantly between patients a variable representing the deviation from the visit-specific mean time will be added to the model. Standard diagnostic tools will be used will be used to assess model fit.

Secondary Endpoints

We will determine whether RARC is superior to ORC in terms of blood loss. More specifically, the overall blood loss due to surgery will be compared between the two treatment arms. In the ORC group the average blood loss is 575 ml [16]. 288 patients yield 90% power to detect a difference of blood loss between the two treatment groups of 20%. These calculations are based on the assumption that the amount of blood loss is normally distributed, that the average blood loss in the open surgery group is 575ml, and that the standard deviation of blood loss is 300ml. A one-sided significance level of 0.025 was used.

We will determine whether RARC is superior to ORC in terms of transfusion rates. The transfusion rates (proportion of patients requiring blood transfusions) will be compared between the two treatment arms. The transfusion rate for ORC is approximately 75%(from Table 2, unpublished). 288 patients yield 92% power and a one-sided significance level of 0.025 to detect a difference of transfusion rates between arms of at least 20%. These calculations are based on the assumption that the transfusion rate is binomially distributed.

Table 2 (Preliminary Data from University of Texas Health Sciences Center, San Antonio)

	ORC (n=12)	*RARC(n=12)*	*p-value*
Median (IQR) Units of Blood Given	*2.5 (1-5)*	*0 (0-3)*	*0.082*
Transfusion given (%)	*9/12 (75%)*	*4/12 (33%)*	*0.041*
Median (IQR) LOS (days)	*6.5 (6-8.5)*	*6.5 (5-9.5)*	*0.554*
LOS (5 days or less)	*0/12 (0%)*	*4/12 (33.3%)*	*0.028*
Median No. LNs (IQR)	*19 (6-27)*	*10.5 (8.5-17.5)*	*0.30*
Positive margin	*3/12 (25%)*	*0/12 (0%)*	*0.064*

All positive margins in the ORC group had pT4 disease.

Length of hospital stay will be used as a surrogate for recovery after surgery. We will determine whether RARC is superior to ORC in terms of length of hospital stay. Currently, all of the patients receiving ORC stay in the hospital for more than 5 days while 67% of patients in the RARC group stay in the hospital for more than 5 days (from Table 3, unpublished). 288 patients yield 97% power and a one-sided significance level of 0.025 to detect a difference in percent of patients requiring a hospital stay beyond 5 days between RARC and ORC arms of at least 20%. These calculations are based on the assumption that the percent patients requiring a hospital stay beyond 5 days is binomially distributed.

Table 3 (Preliminary Data for Cost Analyses from University of North Carolina)

	ORC (n=21)	RARC (n=20)	P VALUE
Age (median)	70	70	
OR time (mins)	293	389	<0.001
OR fees (dollars)	5441	6202	<0.00001
OR disposables (dollars)	2485	3715	0.0003
OR capital + reusables	50	2000	
LOS (days)	6	4	0.02 (Mann-Whitney U)
Room and Board	5954	3664	0.005 (Mann-Whitney U)
Overall Costs	19047	19837	0.14(Mann-Whitney U)

Hand Grip Strength at 3 months after surgery will be measured as a surrogate for recovery after surgery. It was found that only 39% of patients recovered at three months after a major abdominal surgery as measured by the Hand Grip Strength[14].We will compare the proportion of patients recovered as measured by Hand Grip Strength between the two treatment arms. We hypothesize that 20% more patients will have recovered three months after surgery in the RARC arm compared to the ORC arm. A total of 288 patients yield 91% power and a one-sided significance level of 0.025 to detect a difference between arms of at least 20%. These calculations are based on the assumption that Hand Grip Strength at three months is binomially distributed.

Surgical margin as a measure for local cancer control will be measured as positive or negative for each patient and compared between arms using a Fisher's exact test. The number of nodes resected in each arm will be compared using a t-test.

Progression-free and overall survival will be evaluated using the method of Kaplan Meyer and comparisons between arms will be made using the stratified log-rank test.

All efficacy and QL endpoints will be assessed using the PP population.

Analysis Populations:

Per-Protocol Population (PP Population):

The per-protocol population includes all patients who have met inclusion/exclusion criteria and received the surgery to which they were randomized. This is the primary efficacy population. All efficacy and QL endpoints will be assessed using this population.

Intent-to-Treat Population (ITT Population):

The ITT population includes all patients who have been randomized to the trial. Patients are assigned to treatment arms based on what they are "randomized" to receive. This is the sensitivity analysis population of the primary efficacy endpoint.

6. HUMAN SUBJECTS

Compensation

There will be no compensation provided to subjects for participating in the study.

Risks to Subjects

The only research related risks to subjects are the potential loss of PHI and potential mental distress during conduct of questionnaires. Measures to maintain confidentiality are being employed. For a detailed description, please see the section titled “Confidentiality.” Should patients express discomfort during questionnaires or other assessments, they will first be allowed to take a break from questioning. If distress persists, the session will be terminated. Participants may refuse to answer questions which cause them discomfort rather than being withdrawn from the study.

Special Precautions

Subject data will be examined at each follow up visit and subjects queried for adverse events (AE) defined as complications related to the robotic/open cystectomy and/or study procedures. An AE or complication is the appearance of undesirable sign(s), symptom(s), or medical condition(s) occurring after a participant signs the informed consent and considered to be related to the robotic/open cystectomy and/or study procedure. A serious adverse event is any untoward medical occurrence that:

5. Is fatal or life-threatening
6. Requires hospitalization or prolongation of existing hospitalization
7. Results in disability/incapacity
8. Is medically significant in that it may jeopardize the patient and may require medical or surgical intervention to prevent one of the outcomes listed above.

All AEs or complications will be graded for severity according to the modified Clavien grading system. All adverse events will be reported to the IRB at the time of annual review and to the DSMC as described below.

The investigator is obligated to assess the relationship between any study-related procedure and the occurrence of each SAE. The investigator will use clinical judgment to determine the relationship. Alternative causes, such as natural history of the underlying diseases, concomitant therapy, other risk factors, and the temporal relationship of the event to any study-related procedure will be considered and investigated.

Even in situations when an SAE has occurred and the investigator has incomplete information to include in the initial SAE report, the investigator will make an assessment of causality for every event prior to reporting it. The investigator may change his opinion of causality in light of follow-up information and amend the SAE case report form and report accordingly.

SAEs meeting the IRB definition of Unanticipated Problems Involving Risk to Subjects or others (UPIRSO) will be reported to the IRB within 7 days, and within 48 hours if life-threatening or fatal or will follow the guidelines as required by the local IRBs.

All SAEs will also be reported to the coordinating center office of Dr. Parekh at the University of Miami. After completion of review by Dr. Parekh, SAEs will be summarized and communicated across sites via the posting to the study website at https://prodq.crab.org/Parekh/Login.aspx. The data will be reviewed on a biweekly basis by the investigators to ensure quality control and safety. During the study when there is a safety evaluation, the investigator and/or research staff will be responsible for detecting, documenting and reporting any adverse events or serious adverse events to the IRB.

Subject Completion and Withdrawal

A subject will be considered completed when he/she has completed all follow-up visits up to the 24-month evaluation. A subject may discontinue participation in this study at any time at the investigator's discretion or at the request of the subject. The reason for study withdrawal will be documented in the study related source documentation.

Alternative Treatments

Subjects who are eligible for the proposed study will be randomized to receive either open or robotic-assisted cystectomy. The alternative to participating in this study would be for a subject to choose which surgical technique will be used rather than being randomized to one or the other.

Confidentiality

Maintaining confidentiality of patient-specific information will be top priority throughout all

phases of the study. Patient data (PHI) will be compiled in a database and de-identified upon completion of analysis. Cases in the database will be identified by initials, patient identification number, the year of patient's birth, and the date of surgery. The database will not include information which can be identifiable with the link (or key). All electronic data will be stored in a password protected database in accordance with institutional computer-security policies. The identification number is actually a research record number that cannot be linked to the subject except by a key. This key will be maintained securely by the Principal Investigator. The data will be stored and maintained by the CRAB informatics core facility.

Data Safety Monitoring Committee

A Data and Safety Monitoring Board will oversee the conduct of the study. The Board consists of 5 voting, independent members: 1 surgeon, 1 medical oncologist, 1 CCRA/RN, 1 biostatistician, and 1 lay person. Non-voting members include support staff from Cancer Research And Biostatistics (who will prepare the DSMC reports), and project faculty (Principal Investigators) as appropriate. DSMC members receive database summaries from CRAB, including adverse events and post-surgical complications reports, serious adverse event summaries, and other pertinent patient/treatment summary information. Meetings occur every six months, convened via teleconference. The DSMC is responsible for decisions regarding possible termination and/or early reporting of the study.

7. LITERATURE CITED

1. Jemal A, Siegel R, Ward E, et al. Cancer statistics, 2008. CA: a cancer journal for clinicians 2008;58(2):71-96.
2. Stein JP, Lieskovsky G, Cote R, et al. Radical cystectomy in the treatment of invasive bladder cancer: long-term results in 1,054 patients. J Clin Oncol 2001;19(3):666-75.
3. Stein JP, Skinner DG. Results with radical cystectomy for treating bladder cancer: a 'reference standard' for high-grade, invasive bladder cancer. BJU international 2003;92(1):12-7.
4. Cookson MS, Chang SS, Wells N, Parekh DJ, Smith JA, Jr. Complications of radical cystectomy for nonmuscle invasive disease: comparison with muscle invasive disease. The Journal of urology 2003;169(1):101-4.
5. Shabsigh A, Korets R, Vora KC, et al. Defining Early Morbidity of Radical Cystectomy for Patients with Bladder Cancer Using a Standardized Reporting Methodology. European urology 2008.
6. Konety BR, Allareddy V, Herr H. Complications after radical cystectomy: analysis of population-based data. Urology 2006;68(1):58-64.
7. Stein JP, Skinner DG. Surgical atlas. Radical cystectomy. BJU international 2004;94(1):197-221.
8. Menon M, Hemal AK, Tewari A, et al. Robot-assisted radical cystectomy and urinary diversion in female patients: technique with preservation of the uterus and vagina. Journal of the American College of Surgeons 2004;198(3):386-93.
9. Gill IS. Laparoscopic radical nephrectomy for cancer. The Urologic clinics of North

America 2000;27(4):707-19.

10. Dillenburg W, Poulakis V, Skriapas K, et al. Retroperitoneoscopic versus open surgical radical nephrectomy for large renal cell carcinoma in clinical stage cT2 or cT3a: quality of life, pain and reconvalescence. European urology 2006;49(2):314-22; discussion 22-3.

11. Wang GJ, Barocas DA, Raman JD, Scherr DS. Robotic vs open radical cystectomy: prospective comparison of perioperative outcomes and pathological measures of early oncological efficacy. BJU international 2008;101(1):89-93.

12. Guru KA, Kim HL, Piacente PM, Mohler JL. Robot-assisted radical cystectomy and pelvic lymph node dissection: initial experience at Roswell Park Cancer Institute. Urology 2007;69(3):469-74.

13. Dasgupta P, Rimington P, Murphy D, Elhage O, Challacombe B, Khan MS. Robotically assisted radical cystectomy. BJU international 2008;101(12):1489-90.

14. Lawrence VA, Hazuda HP, Cornell JE, et al. Functional independence after major abdominal surgery in the elderly. Journal of the American College of Surgeons 2004;199(5):762-72.

15. Fitzmaurice GM LN, Ware JH. . Applied longitudinal analysis. NY. John Wiley & Sons 2004.

16. Probstfield JL, Wittes JT, Hunninghake DB. Recruitment in NHLBI population-based studies and randomized clinical trials: data analysis and survey results. Controlled clinical trials 1987;8(4 Suppl):141S-9S.

17. Moyé L. Statistical monitoring of clinical trials:. Fundamentals for investigators 2006.;NY: Springer.

Appendix A: ECOG [a] Performance Status Scale

Grade	Status
0	Fully active, able to carry on all pre-disease performance without restriction
1	Restricted in physically strenuous activity but ambulatory and able to carry out work of a light or sedentary nature, eg, light house work, office work
2	Ambulatory and capable of all selfcare but unable to carry out any work activities. Up and about more than 50% of waking hours
3	Capable of only limited selfcare, confined to bed or chair more than 50% of waking hours
4	Completely disabled. Cannot carry on any selfcare. Totally confined to bed or chair
5	Dead

[a] As published in *Am J Clin Oncol (CCT)*. 1982;5:649-655.

Summary of Changes

Date	Changes Made
11/1/2011	• Revised study sites, update Off-site Research Sites<br>• Updated study sites, update Off-site Research Sites<br>• Updated Name and Title of Co-Investigator and Title of Consultant, Co-Investigator change and Consultant title change<br>• Moved text from Specific Aim 1 Item 1 to Specific Aim 2 Items 6, clarification purposes<br>• Stated length of study duration, clarification purposes<br>• Defined Progression Free Survival and Death, clarification purposes<br>• Updated Table 1: Study Calendar to include assessment ECOG Performance Status and added study windows of +/- 2 weeks at Hospital Discharge for OR & Hospital Costs and +/- 30 days at 4-6 weeks, 3 months, and 6 months for Activities of Daily Living (ADL) score, Instrumental Activities of Daily Living (IADL) score, Hand Grip Strength Test, and Timed Up and Go Walking Test, and +/- 30 days at 3 months and 6 months for Quality of Life Questionnaire (QOL), Vanderbilt Cystectomy Index, and SF8 assessments, inadvertently left out of previous protocol<br>• Added stratification parameter and study duration, clarification purposes<br>• Added Table 2 and corrected text to appropriately reference Tables 2 and 3, inadvertently left out of previous protocol<br>• Defined survival, clarification purposes<br>• Defined SAE reporting procedures, clarification purposes<br>• Added 2 members to Data Safety Monitory Board, safety assessment
12/1/2011	• Updated study sites<br>• Revise Specific Aim 1 Item 2, inadvertently left out of original protocol<br>• Removed mEQ for clarification purposes<br>• Updated Table 1 for clarification purposes
1/4/2012	• Re-defined Progression Free Survival for clarification purposes<br>• Re-defined Overall Survival for clarification purposes.<br>• Revised Statistical Considerations section for clarification purposes

5/1/2012	• Updated Dr. Parekh’s title and affiliation<br>• Removed “local sites” and listed all sites as “research sites”, UTHSCSA no longer coordinating IRB<br>• Updated Dr. Parekh’s title<br>• Revised number of study sites to 15<br>• Revised Inclusion Criteria #2 to include Refractory Cis<br>• Revised number of study sites to 15<br>• Revised location of SAE report to be sent to University of Miami
10/1/2012	• Updated Table 1: Study Calendar, clarification purposes
6/16/2013	• Updated Responsible Entities and Staff Titles and Affiliations
3/6/2014	• Increase total enrollment to approximately 350 (approximately 175 participants in each arm) to ensure 288 evaluable participants’ data is available for analysis.<br>• Define evaluable participant for clarification purposes.<br>• Increase total enrollment to approximately 350 (approximately 175 participants in each arm) to ensure 288 evaluable participants’ data is available for analysis.<br>• Section titled “Analysis of Primary Endpoint”, 2nd paragraph, delete last sentence. Paragraph was added in previous version of protocol and this sentence was inadvertently not deleted.<br>• Section titled “Per Protocol Population”, delete 1st sentence and define surgery to which participants are randomized, clarification purposes.
