# Supplementary material for: Comparison of Robot-Assisted and Open Radical Cystectomy in Recovery of Patient-Reported and Performance-Related Measures of Independence: A Secondary Analysis of a Randomized Clinical Trial
Source: JAMA Netw Open. 2022 Feb 16;5(2):e2148329. doi: 10.1001/jamanetworkopen.2021.48329 (PMC8851298; doi:10.1001/jamanetworkopen.2021.48329)
Supplement: Supplement 2. — eFigure 1. ADL, iADL, TUGWT, and HGS Summary Scores of the Entire Cohort eFigure 2. ADL, iADL, TUGWT, and HGS Summary Scores by Surgical Approach (Study Groups) eFigure 3. ADL, iADL, TUGWT, and HGS Summary Scores by Type of Urinary Diversion eFigure 4. RAZOR Trial CONSORT Flow Diagram eTable. Recovery of HGS and ADL at 1 and 3 Months After Surgery [file jamanetwopen-e2148329-s002.pdf]

Supplementary Online Content

Venkatramani V, Reis IM, Gonzalgo ML, et al. Comparison of robot-assisted and open radical cystectomy in recovery of patient-reported and performance-related measures of independence: a secondary analysis of a randomized clinical trial. *JAMA Netw Open*. 2022;5(2):e2148329. doi:10.1001/jamanetworkopen.2021.48329

eFigure 1. ADL, iADL, TUGWT, and HGS Summary Scores of the Entire Cohort
eFigure 2. ADL, iADL, TUGWT, and HGS Summary Scores by Surgical Approach (Study Groups)
eFigure 3. ADL, iADL, TUGWT, and HGS Summary Scores by Type of Urinary Diversion
eFigure 4. RAZOR Trial CONSORT Flow Diagram
eTable. Recovery of HGS and ADL at 1 and 3 Months After Surgery

This supplementary material has been provided by the authors to give readers additional information about their work.

© 2022 Venkatramani V et al. *JAMA Network Open*.

eFigure 1. ADL, iADL, TUGWT, and HGS Summary Scores of the Entire Cohort

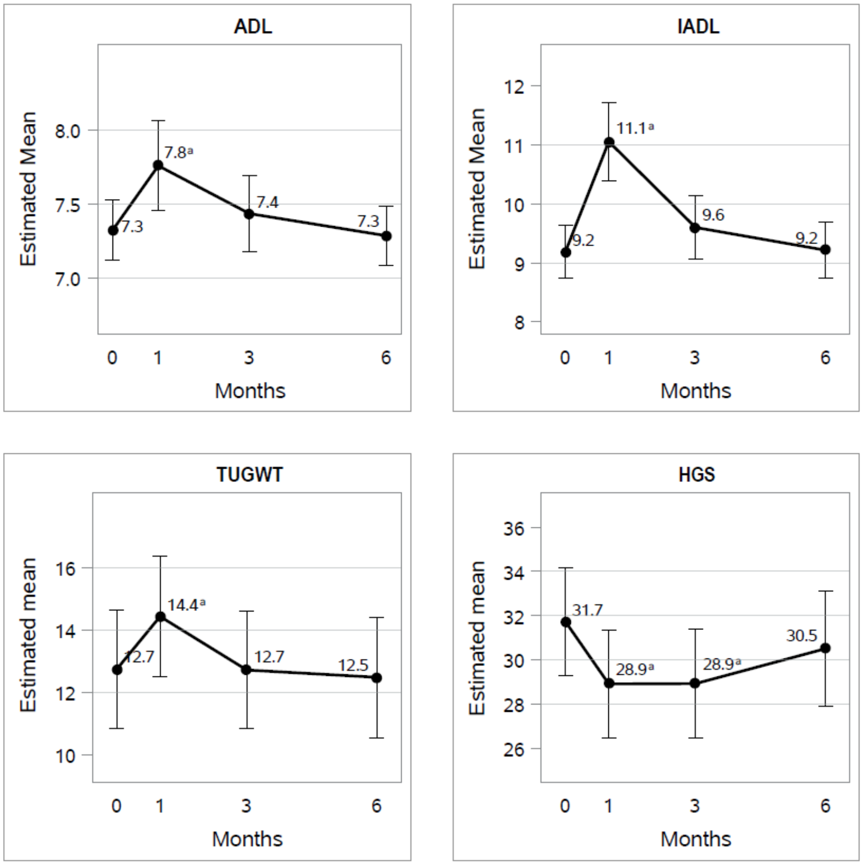

Plots show estimated mean score and corresponding 95% confidence intervals from mixed models including time, arm, diversion, time×arm and time×diversion interactions, age, sex, BMI, ECOG PS, T-stage, and perioperative chemotherapy, and accounting for site in the random effect component of the model. [a] p≤0.05: Mean difference compared with baseline, statistically significant at the 5% significance using Bonferroni's adjustment for multiple comparisons.

© 2022 Venkatramani V et al. *JAMA Network Open*.

eFigure 2. ADL, iADL, TUGWT, and HGS Summary Scores by Surgical Approach (Study Groups)

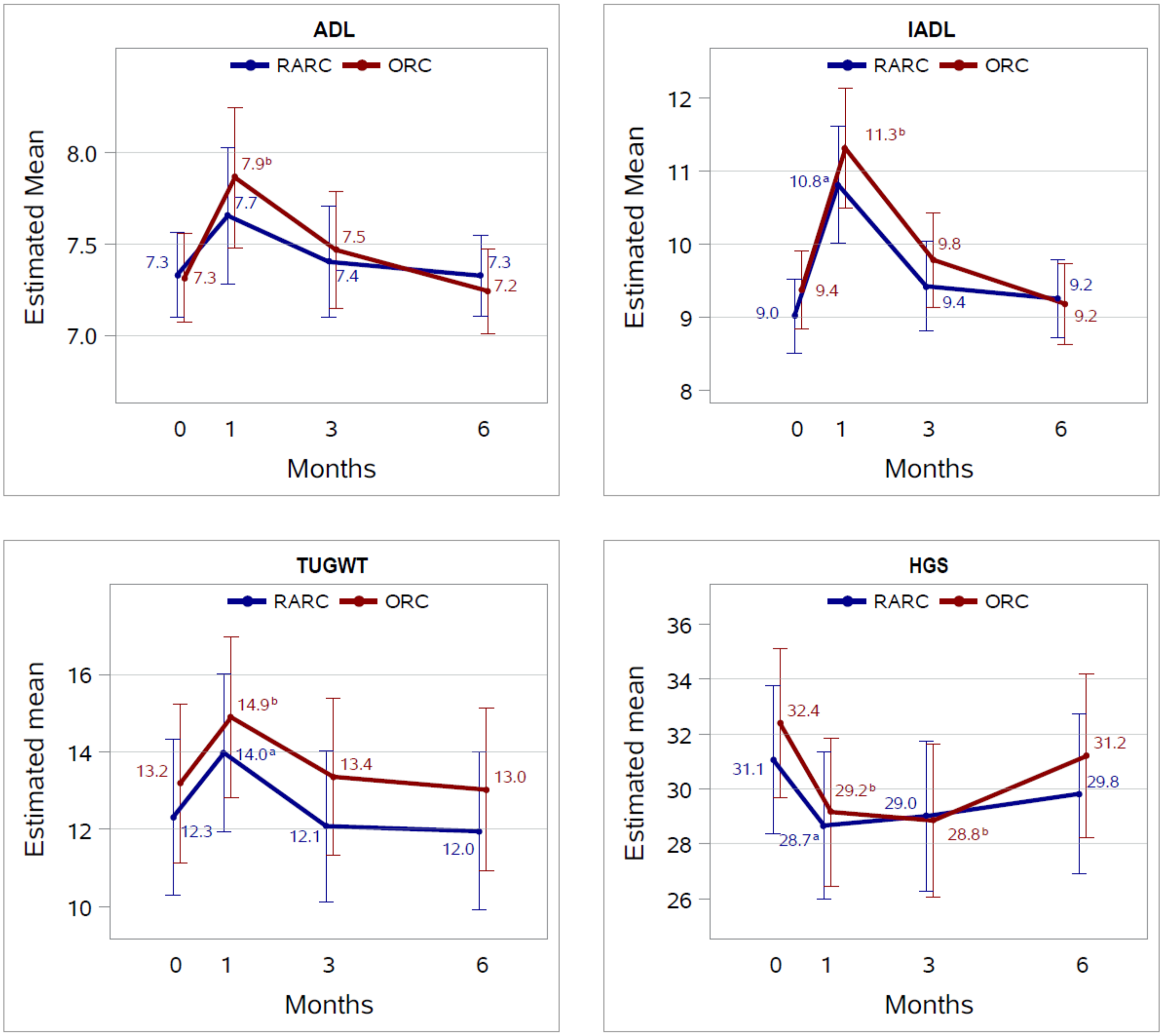

Plots show estimated mean score and corresponding 95% confidence intervals from mixed models including time, arm, diversion, time×arm and time×diversion interactions, age, sex, BMI, ECOG PS, T-stage, and perioperative chemotherapy, and accounting for site in the random effect component of the model. [a] p≤0.05: Mean difference compared with baseline, statistically significant at the 5% significance using Bonferroni's adjustment for multiple comparisons.

© 2022 Venkatramani V et al. *JAMA Network Open*.

eFigure 3. ADL, iADL, TUGWT, and HGS Summary Scores by Type of Urinary Diversion

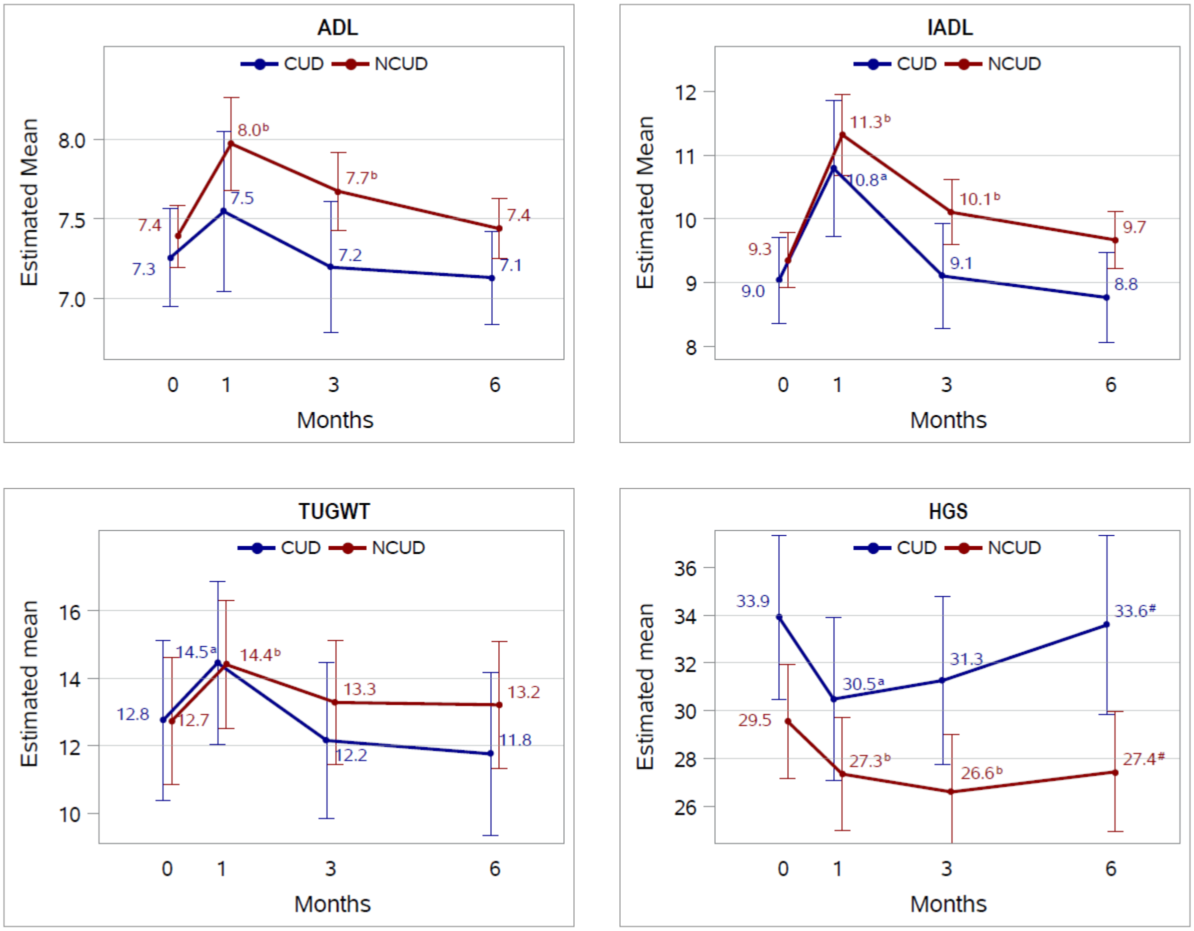

Plots show estimated mean score and corresponding 95% confidence intervals from mixed models including time, arm, diversion, time×arm and time×diversion interactions, age, sex, BMI, ECOG PS, T-stage, and perioperative chemotherapy, and accounting for site in the random effect component of the model. [a] p≤0.05: Mean difference compared with baseline, statistically significant at the 5% significance using Bonferroni's adjustment for multiple comparisons.

© 2022 Venkatramani V et al. *JAMA Network Open*.

eFigure 4. RAZOR Trial CONSORT Flow Diagram

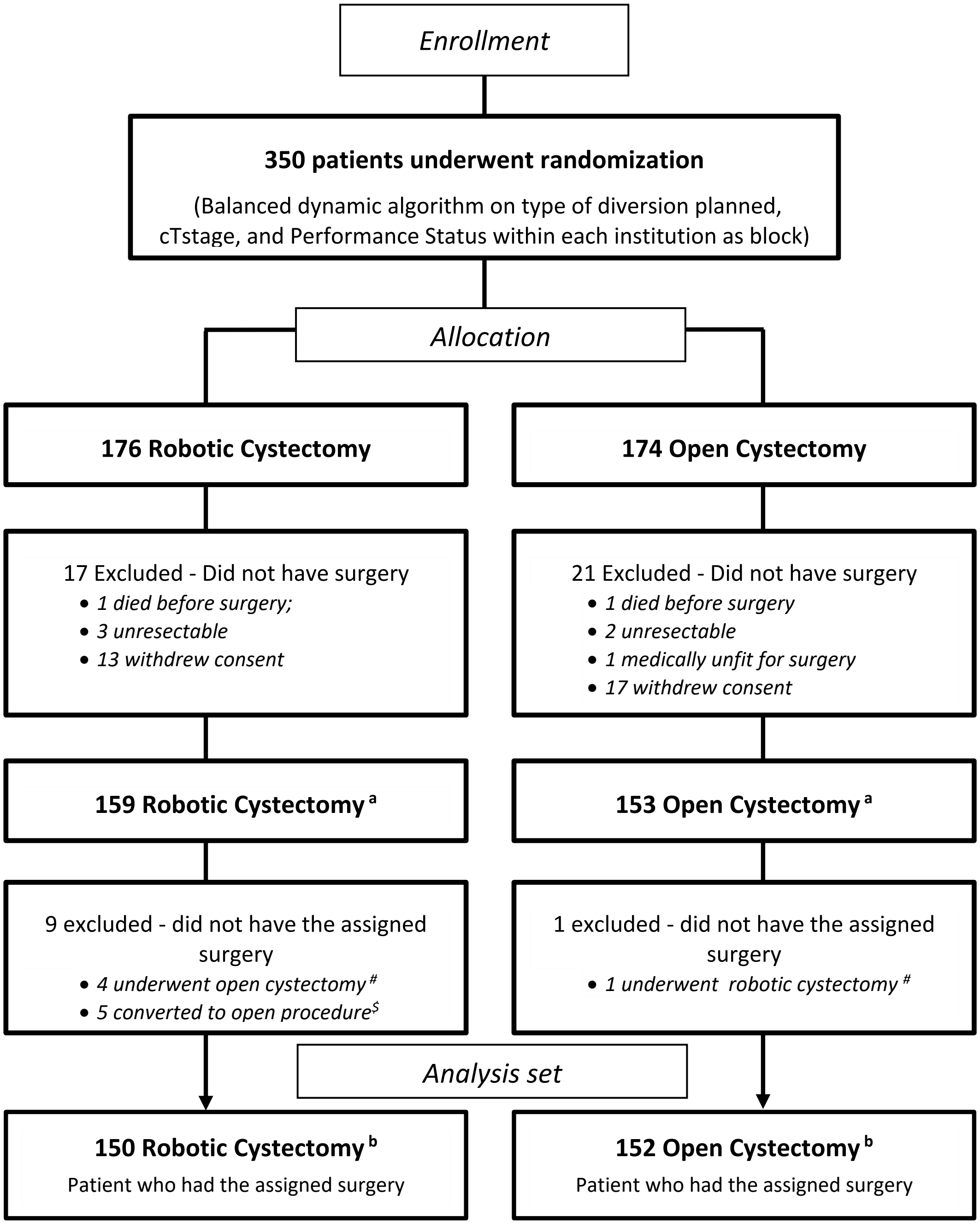

Note: The number assessed for eligibility is not available. Not all "per protocol" patients were included in analyses of each outcome.

[#] Patients received the wrong procedure due to screening failure (n=2), failure to notify surgeon of randomization (n=2), and unknown reasons (n=1).

[$] Conversions were due to locally advanced disease (n=2), inadequate visualization (n=1), inability to tolerate steep Trendelenburg position (n=1) and incidentally detected coexisting large colonic mass (n=1).

[a] This constitutes the *modified intention-to-treat* population used on the sensitivity analysis of the primary endpoint 2-year progression-free survival.

[b] This constitutes the *per-protocol* population used in all data analysis. In the 2-year follow-up period, there were 10 and 14 censored observations in robotic and open groups, including 1 and 6 losses to follow-up, respectively.

© 2022 Venkatramani V et al. *JAMA Network Open*.

eTable. Recovery of HGS and ADL at 1 and 3 Months After Surgery

	Total N	Recovery of HGS, n (%)		P value
		1 month after surgery		
		Not Recovered	Recovered	
RARC	104	65 (62.5%)	39 (37.5%)	0.423
ORC	112	64 (57.1%)	48 (42.9%)	
		3 months after surgery		
		Not Recovered	Recovered	
RARC	90	58 (64.4%)	32 (35.6%)	0.911
ORC	88	56 (63.6%)	32 (36.4%)	
	Total N	Recovery of ADL, n (%)		P value
		1 month after surgery		
		Not Recovered	Recovered	
RARC	123	25 (20.3%)	98 (79.7%)	0.486
ORC	125	30 (24.0%)	95 (76.0%)	
		3 months after surgery		
RARC	109	11 (10.1%)	98 (89.9%)	0.596
ORC	105	13 (12.4%)	92 (87.6%)	

Note: The measurement of Hand Grip Strength (HGS) at 3 months after surgery was the primary endpoint for assessing recovery, with patients categorized as “not recovered” or “recovered” to (or better than) preoperative HGS. Recovered on ADL was defined as an ADL value greater or equal to preoperative ADL value.

© 2022 Venkatramani V et al. *JAMA Network Open*.
