# Supplementary material for: Comparison of Robot-Assisted and Open Radical Cystectomy in Recovery of Patient-Reported and Performance-Related Measures of Independence: A Secondary Analysis of a Randomized Clinical Trial
Source: JAMA Netw Open. 2022 Feb 16;5(2):e2148329. doi: 10.1001/jamanetworkopen.2021.48329 (PMC8851298; doi:10.1001/jamanetworkopen.2021.48329)
Supplement: Supplement 3. — Data Sharing Statement [file jamanetwopen-e2148329-s003.pdf]

Data Sharing Statement

Venkatramani. Comparison of Robot-Assisted and Open Radical Cystectomy in Recovery of Patient-Reported and Performance-Related Measures of Independence. *JAMA Netw Open*. Published February 16, 2022. doi:10.1001/jamanetworkopen.2021.48329

Data
Data available: Yes
Data types: Deidentified participant data
How to access data: ireis@med.miami.edu
When available: With publication

Supporting Documents
Document types: None

Additional Information
Who can access the data: Approved researchers
Types of analyses: Specified purpose
Mechanisms of data availability: After approval of a proposal
